# Synthesis of a *fac*-Tricarbonylrhenium(I) Complex with Pyrithione, Its Physicochemical Characterization, and Assessment of Biological Effects

**DOI:** 10.1021/acsomega.5c06647

**Published:** 2025-08-15

**Authors:** Uroš Rapuš, Tamás Pivarcsik, Ana Mitrović, Jakob Kljun, Anita Bogdanov, Gabriella Spengler, Anže Meden, Stanislav Gobec, Janko Kos, Éva A. Enyedy, Iztok Turel

**Affiliations:** † Faculty of Chemistry and Chemical Technology, 37663 University of Ljubljana, Večna pot 113, SI-1000 Ljubljana, Slovenia; ‡ Department of Molecular and Analytical Chemistry, Interdisciplinary Excellence Centre, 37442 University of Szeged, Dóm tér 7-8., H-6720 Szeged, Hungary; § Faculty of Pharmacy, University of Ljubljana, Aškerčeva cesta 7, SI-1000 Ljubljana, Slovenia; ∥ Department of Biotechnology, Jožef Stefan Institute, Jamova 39, SI-1000 Ljubljana, Slovenia; ⊥ Department of Medical Microbiology, Albert Szent-Györgyi Health Center and Albert Szent-Györgyi Medical School, University of Szeged, Semmelweis u. 6, H-6725 Szeged, Hungary

## Abstract

Research on rhenium complexes containing *fac*-tricarbonyl fragments has been on the rise in recent decades. Some complexes of this type exhibit advantageous properties that can be utilized in diagnostic and therapeutic applications. Herein, we report on the synthesis, structural characterization, solution speciation, and biological activity with mode of action studies of a new *fac*-tricarbonylrhenium­(I) complex with pyrithione ligand *fac*-[Re­(CO)_3_(pyrithionato)­(benzonitrile)] (**4**). In an attempt to prepare a stable rhenium­(I) complex with a pyrithionato ligand, several synthesis procedures were investigated and various products were discovered. In solution, the monodentate benzonitrile ligand can be replaced by a coordinating solvent molecule; however, the carbonyl ligands and pyrithione remain bound to the rhenium center. Complex **4** exhibited strong cytotoxic and antibacterial activity and effectively inhibited the growth of the HSV-2 virus. Additionally, complex **4** was also able to inhibit the cathepsin B enzyme (both its endo- and exopeptidase activities). *In silico* experiments confirmed that complex **4** can interact with cathepsin B near its active site, which may contribute to reduced enzymatic activity.

## Graphical abstract

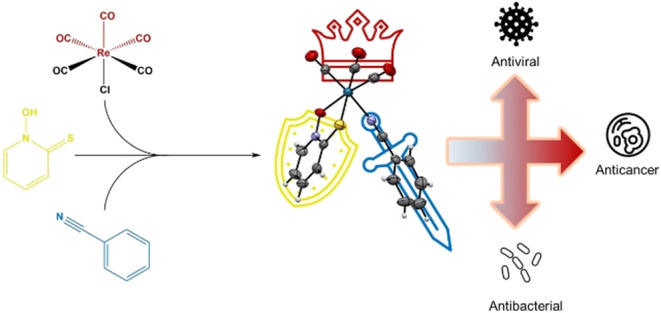

## Introduction

Complexes containing a *fac*-tricarbonylrhenium­(I) core (*fac*-[Re­(CO)_3_]^+^) have gained increasing attention in recent years due to their favorable spectroscopic properties and versatile biological activity. Many rhenium complexes have long-lived emission states, high quantum yields, and can be easily followed in cells with emission microscopy.^1–3^ Furthermore, rhenium complexes can also be used in radioimaging and therapy with radioactive rhenium isotopes or technetium analogues due to their similarity in the coordination chemistry.^4^ Biological activities include antitumor,^3,5–18^ antibacterial,^3,9,17,19–24^ antiviral,^25,26^ and antiparasitic^27^ properties. The reported complexes have been shown to exert their pharmacological effects through interaction with multiple biological targets. While this may pose a challenge in drug development, it may also provide an advantage by reducing the likelihood of drug resistance.^28^

Pyrithione (pthH) is a natural compound produced enzymatically from amino acid precursors in Persian shallot (*Allium stipitatum*). This shallot has long been used in Asia as a spice and in traditional medicine.^29^ Pyrithione has been the subject of scientific research for almost 75 years.^30^ After its discovery, complexes with various metals were soon prepared, some of which showed interesting biological activities. The two most successful complexes were copper­(II) and zinc­(II) bispyrithionato complexes, also known as copper pyrithione and zinc pyrithione. Due to their broad-spectrum antimicrobial properties, both are used in antifouling paints, and zinc pyrithione is also used in antidandruff shampoos. This use was banned in the EU in 2022 due to environmental concerns but is still used in the rest of the world.^31^ Pyrithione complexes are known for most metals in the periodic table. In most cases, the complexes are neutral compounds with simple binary [M^II^(pth)_2_] or [M^III^(pth)_3_] structures. This results in their poor aqueous solubility, which can be useful for some specific applications (shampoos and paints mentioned above), but generally, a good aqueous solubility is essential for drugs. It was found that certain ternary pyrithione complexes such as the pseudooctahedral organoruthenium­(II) [ruthenium­(η^6^-*p*-cymene)­(pyrithionato)­(chlorido)] complex ([Ru­(cym)­(pth)­Cl]) (Figure 1) and its analogues are more soluble in water and show promising biological activities.^32^

**Figure 1:**
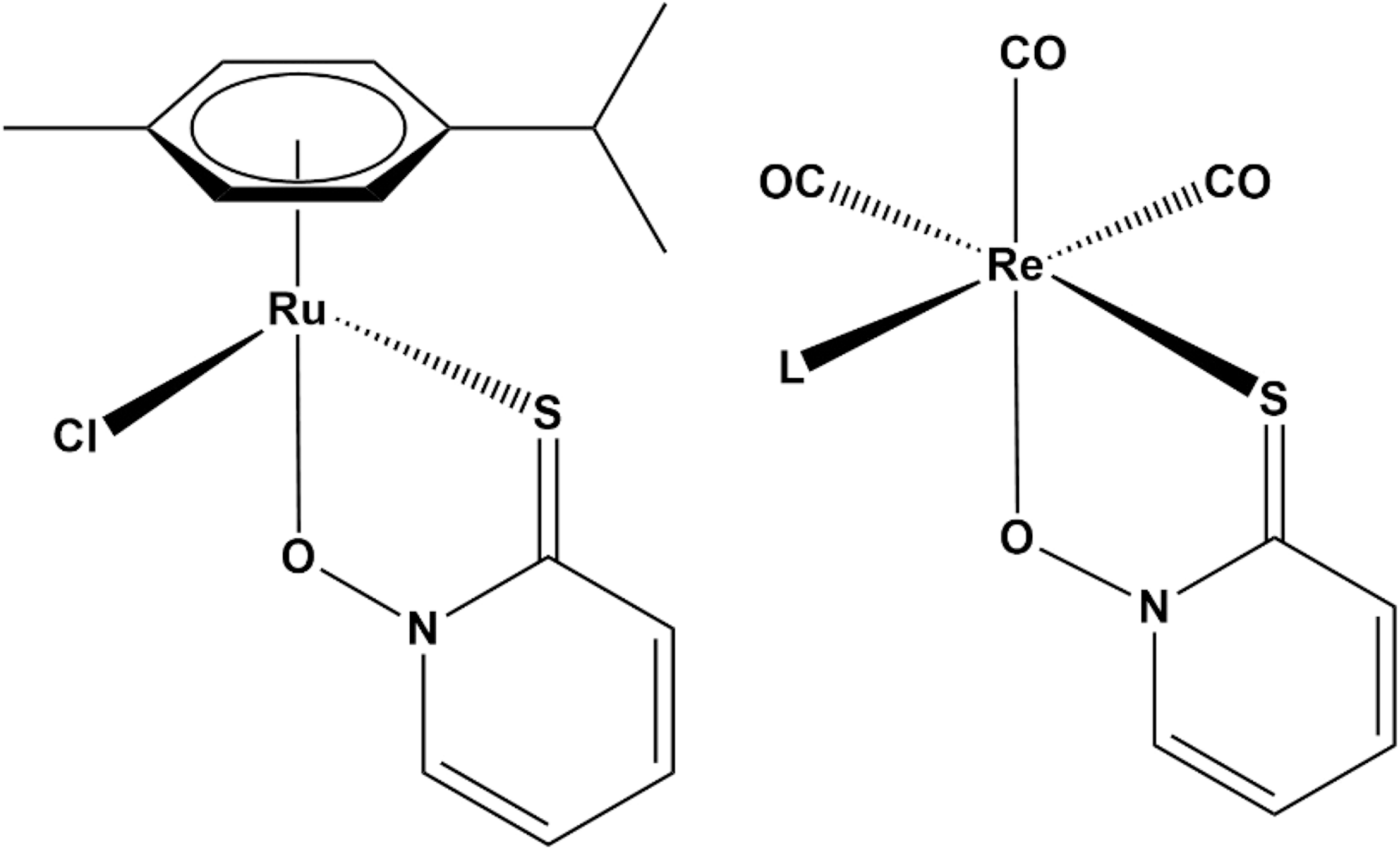
[Ru­(cym)­(pth)­Cl] (left) and structure of desired *fac*-[Re­(CO)_3_(pth)­L] complex (right).

In recent years, we have developed metal complexes with various pyrithione analogues and investigated their biological properties. We studied the anticancer and antiviral activities of organoruthenium­(II) and zinc­(II) complexes with pyrithione ligands and explored some details of their mechanisms of action. Antitumor and antiviral activities of organoruthenium­(II) complexes were demonstrated through the inhibition of lysosomal cysteine peptidases including cathepsin B.^32–36^ The results of the studies showed good potential of pyrithiones as ligands in metal-based drugs, so we decided to extend our research to rhenium­(I) compounds due to their previously mentioned favorable spectroscopic and pharmacological properties. The chemistry of rhenium with pyrithione is practically unexplored as only one rhenium complex with pyrithione is reported in the literature. This is an oxorhenium­(V) complex with a monodentate sulfur-bound pyrithione ligand and a completely different coordination sphere.^37^

The aim of our study was to prepare a rhenium complex with a structure similar to that of [Ru­(cym)­(pth)­Cl]. The compound should be a sufficiently stable octahedral rhenium­(I) complex with the *fac*-tricarbonyl fragment mimicking the arene-ruthenium species, an *O*,*S*-bound bidentate pyrithionato ligand and one neutral monodentate ligand L (*N*- or *S*-monodentate ligand) yielding a compound with the general formula (*fac*-[Re­(CO)_3_(pth)­L]) (Figure 1). Several side products that were observed during the synthesis were subsequently isolated and characterized. The stability of complex *fac*-[Re­(CO)_3_(pth)­(benzonitrile)] (Scheme 1, **4**) and the detailed study of its complex equilibria in aqueous media as well as its interaction with human serum albumin (HSA) are also reported. In addition, cytotoxicity on HeLa, Colo205, and Colo320 human cancer cell lines, antibacterial activity against Gram-positive and Gram-negative bacterial strains, and antiviral activity against the *Herpes simplex* type 2 virus were also tested. Drugs with multiple therapeutic effects are particularly important in the case of cancer due to the weakened immune system. Anticancer drugs that also have good antibacterial and antiviral activities may limit the development of dangerous infections.^38^ Furthermore, a possible mechanism of action of inhibiting cysteine cathepsins, which are important promoters of tumor growth and represent potential targets for new anticancer compounds, was also investigated as rhenium complexes were previously proven to inhibit various enzymes from the cathepsin family.^37,39–41^ Additionally, *in silico* studies were also conducted to see how the complex interacts with cathepsin B through docking and molecular dynamics simulation.

**Scheme 1:**
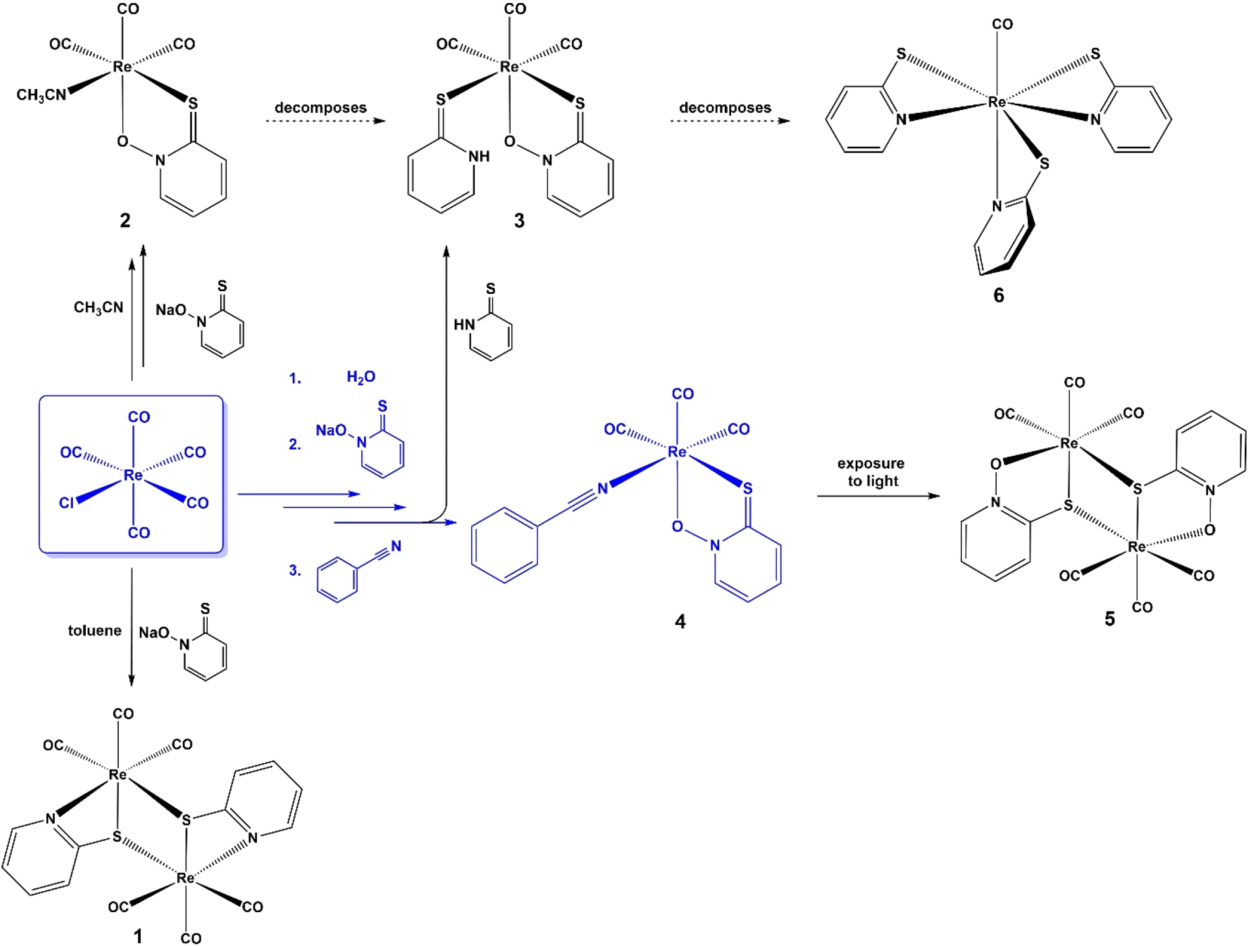
Scheme of Different Compounds Observed While Searching for the Best Route to Prepare Complex **4**

## Results and Discussion

### Synthesis and Characterization of Complexes

In our initial attempt to isolate a rhenium­(I) pyrithionato complex, we reacted rhenium­(I) pentacarbonyl chloride [Re­(CO)_5_Cl] and the sodium salt of pyrithione in toluene at 120 °C for 1 h in sealed high-pressure tubes. The reaction surprisingly resulted in the formation of the previously reported dimeric complex **1** (Scheme 1) with *S*-bridging pyridine-2-thiolato ligands (2-mpy).^42^ Deoxygenation reactions were already observed before in pyrithione as well as in other *N*-oxides.^43,44^ To minimize the possibility of ligand decomposition, we then decided to use milder reaction conditions and first prepared a precursor with labile ligands in place of the carbonyl groups.

The acetonitrile precursor *fac*-[Re­(CO)_3_(acetonitrile)_2_Cl] is used for this purpose in the procedures described in the literature.^45,46^ After formation of the acetonitrile precursor *in situ* and cooling of the reaction mixture, we added sodium pyrithionate and were able to prepare the desired product with the proposed formula *fac*-[Re­(CO)_3_(pth)­(acetonitrile)] (Scheme 1, **2**). Unfortunately, the solid product was not stable after it was removed from solution, as it changed color upon isolation in the presence of air and/or light within minutes. After the solution was stored in the refrigerator, two different types of crystals and precipitate formed. The first type of pale-yellow crystals made up the majority of the crude product. They decomposed quickly after being removed from the solution. The analysis confirmed the structure of expected product **2**. The remaining crystals were orange, and the structure obtained showed the decomposition product **3** with the formula *fac*-[Re­(CO)_3_(pth)­(2-mpyH)] (Scheme 1), in which the monodentate acetonitrile ligand of the unstable product is replaced by a neutral *S*-bound 2-mpyH ligand formed by deoxygenation of pyrithione.

We decided to further modify the reaction conditions by using rhenium trisaqua precursor *fac*-[Re­(CO)_3_(H_2_O)_3_]Cl (*vide infra*) and benzonitrile, which is less volatile and reacts much slower than acetonitrile. The complex **4** *fac*-[Re­(CO)_3_(pth)­(benzonitrile)] (Scheme 1) was finally synthesized in three steps. First, the precursor *fac*-[Re­(CO)_3_(H_2_O)_3_]Cl was prepared according to a slightly modified procedure from the literature.^47^ The precursor was dried, redissolved in methanol, and reacted with sodium pyrithionate. The reaction was carried out in the dark for 16 h at room temperature. In the third step, benzonitrile (4 mol equiv) was added and the reaction mixture was stirred for another day under the same conditions. The precipitate formed was collected by vacuum filtration, and the product was stored in the dark, as it slowly changed color on the surface when exposed to light. The decomposition product was characterized by X-ray diffraction (XRD) on single crystals that appeared from a solution after prolonged standing (in the dark) and was found to be a pyrithionato bridged dimer **5** (Scheme 1).

Additionally, in an attempt to synthesize compound **3**, we utilized the procedure for the synthesis of complex **4** and used 2-mpyH as a coligand instead of benzonitrile. The reaction yielded a yellow compound with multiple sets of peaks in the NMR spectrum. From the NMR sample solution, we were able to crystallize the compound and determined its crystal structure, which revealed an interesting structure of an oxidized 7-coordinated rhenium­(III) complex **6** with the formula [Re­(CO)­(2-mpy)_3_] (Scheme 1). Further NMR analysis of the crystals revealed that the NMR spectrum of the crystals does not match that of the bulk sample, and the crystals are a product of decomposition.

As is clear from the description above, the reactions between the rhenium­(I) precursor and pyrithione are quite complex and dependent on the conditions. However, we were able to reproducibly isolate complex **4**, which is the only compound to satisfy our criteria regarding composition and stability in sufficient quantity for all further experiments. Complex **4** was fully physicochemically characterized and had its biological properties assessed. The complex was analyzed using ^1^H NMR spectroscopy (Figure S1), mass spectrometry (MS), infrared (IR) and ultraviolet–visible (UV–vis) spectroscopy, and elemental analysis (CHN), and since we were able to obtain suitable crystals, X-ray structure analysis was performed. Stability and solution chemical properties of complex **4** were also thoroughly studied by ^1^H NMR and UV–vis spectroscopy. All other complexes were characterized as far as circumstances allowed due to their instability (see Figures S2–S5).

### Crystallization and X-ray Structure Analysis

Single crystals of compounds **1**–**6** were obtained by different crystallization methods either from solutions of pure single compounds or directly from crude reaction mixtures. Crystals of **1** were obtained from a solution in acetone, crystals of **2** and **3** were obtained from a reaction mixture by slowly evaporating acetonitrile in the fridge at 5 °C, crystals of **4** were obtained from a solution in methanol by slowly evaporating the solvent in the dark, crystals of **5** were obtained from a solution in chloroform by slowly evaporating the solvent in the dark, and crystals of **6** were obtained from a solution of NMR sample after slowly evaporating deuterated methanol. The crystal structures of the complexes were determined by single-crystal X-ray diffraction.

All five rhenium­(I) complexes **1**–**5** present an octahedral coordination sphere of the central rhenium­(I) ion as well as three carbonyl ligands in a facial geometry. The structure of **1** (Figure 2) was already reported in the literature.^42^ Structure redetermination was performed with the same measurement conditions as those for the rest of the compounds for better comparison of the results. The structures of **1** and **5** (Figure 2), while to a certain extent similar by presenting a dimeric structure with two 2-mpy and pth ligands, respectively, coordinated to the first ion in a bidentate manner and bridging to the second ion through the sulfur atom. However, they show some notable differences. The most important is obviously a noticeable distortion of the octahedral environment in the case of **1**, which arises from the low bite angle of the anionic 2-mpy ligands and the formation of the strained 4-membered ring upon binding in comparison of the 5-membered ring in the case of the pth ligand. The second major difference is a higher degree of symmetry in the case of **1** evidenced by the nearly equal Re–S bond distances in the case of the chelating and bridging S atom (*d* = 2.54 ± 0.01 Å) (Table 1). On the other hand, in the crystal structure of **5**, the distance to the chelating sulfur atoms is shorter by Δ = 0.10Å (2.46 vs 2.56 Å) (Table 1). This could explain the tendency of the pyrithione dimer to dissociate and form monomers with coordinating solvent molecules such as acetonitrile, benzonitrile, or pyrithione decomposition product 2-mpyH. In the crystal structures of compounds **2** and **4** (Figure 2), the exchange of acetonitrile with benzonitrile does not result in significant changes in bond length or angle changes. The rhenium–sulfur bond lengths are of the same values as in the case of the length in the chelating pth ligand in the structure of dimer **5**. The structure of rhenium­(III) complex **6** (Figure 2) shows the central rhenium­(III) ion with a pentagonal bipyramidal geometry and expectedly differs significantly in terms of bond length values due to the different oxidation state of the rhenium ion. The single carbonyl ligand occupies one axial position, while two equatorial 2-mpy ligands are bound in the *cisoid* conformation. The third, chemically different 2-mpy ligand is bound perpendicularly, occupying the remaining equatorial position (S^3^) (*transoid* to the two other sulfur atoms) and an axial position (N^3^) forming a S^1^N^1^S^3^N^2^S^2^ ring in the equatorial plane. Interestingly, it is the Re–S bond length of the sulfur atom of the axial ligand, which lies in-plane with the other two 2-mpy ligands, that is significantly shorter in comparison to the two other ligands, while the three rhenium–nitrogen bonds are equidistant.

**Figure 2:**
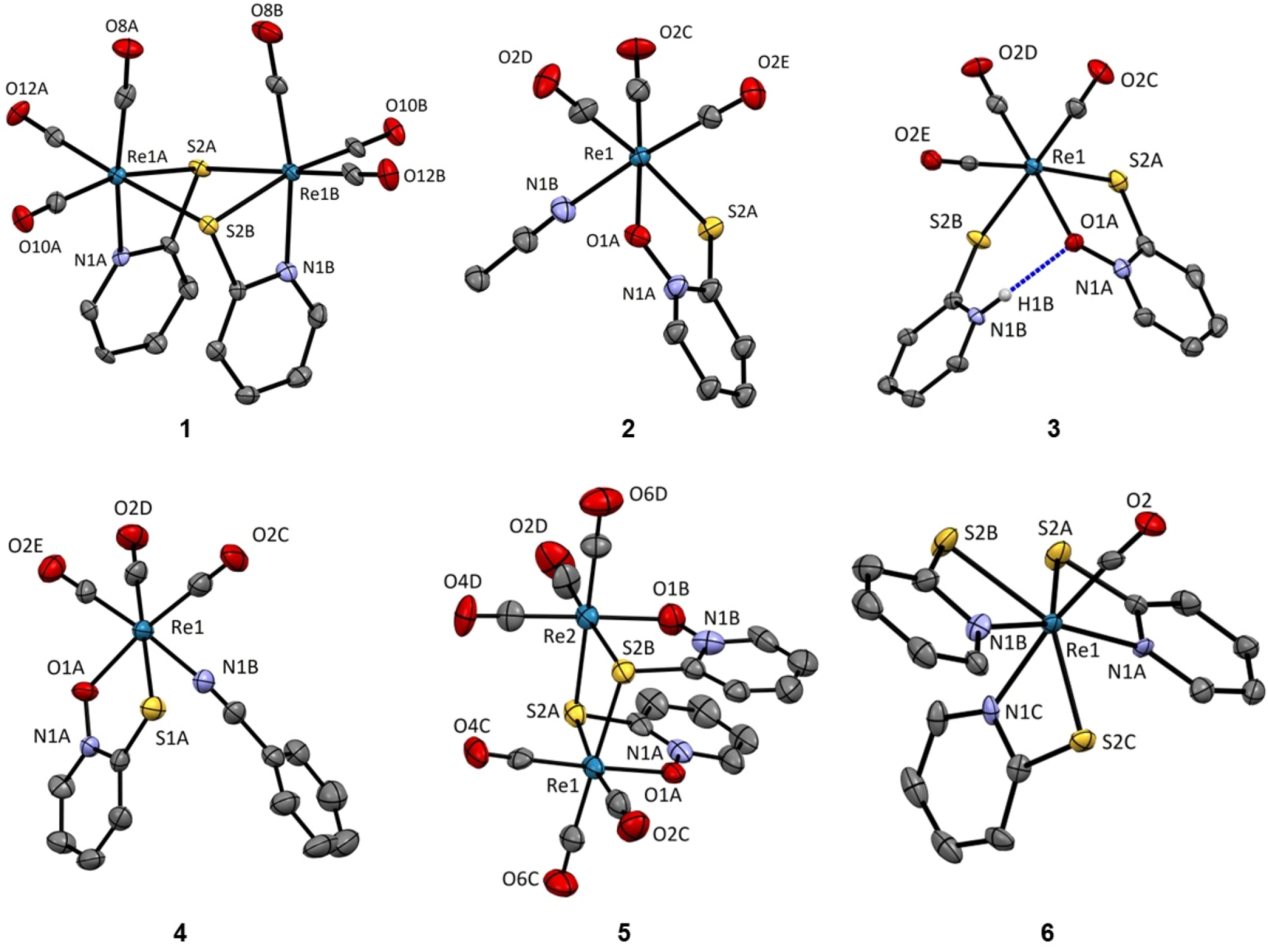
Crystal structures of complexes **1**–**6**. Thermal ellipsoids are drawn at the 50% probability level. All hydrogen atoms, except those bound to heteroatoms, are omitted.

**Table 1:** Selected Bond Lengths in Rhenium Complexes^a^

	**1**	**2**	**3**	**4**	**5**	**6**
Re–C_CO_	1.908(9)				1.950(11)	1.875(11)
	1.935(10)				1.920(13)	
	1.914(10)	1.91(2)	1.918(5)	1.921(10)	1.888(12)	
	1.926(10)	1.88(2)	1.901(5)	1.917(12)	1.932(14)	
	1.939(11)	1.90(2)	1.923(5)	1.911(11)	1.923(12)	
	1.902(10)				1.906(13)	
Re–N_py_	2.153(7)	/	/	/	/	2.189(8)_eq_
	2.140(8)					2.173(8)_eq_
						2.175(8)_ax_
Re–N_nitrile_	/	2.157(17)	/	2.156(9)	/	/
Re–S	2.539(2)_chel_	2.461(5)		2.454(3)_pth_	2.468(3)_chel_	2.492(3)_eq_
	2.548(2)_brdg_		2.4505(13)_pth_		2.565(3)_brdg_	2.499(3)_eq_
	2.532(2)_chel_		2.5342(12)_2mpy_		2.467(3)_chel_	2.406(2)_ax_
	2.546(2)_brdg_				2.568(3)_brdg_	
Re–O_pth_	/	2.109(14)	2.149(3)	2.156(7)	2.122(9)	/
					2.139(8)	

a Abbreviations: py = pyridine, pth = pyrithione, chel = chelating, brdg = bridging, 2mpy = 2-mercaptopyridine, eq = equatorial, ax = axial.

### Stability and Solution Chemical Properties of Complex 4

To investigate the stability of complex **4**, time-dependent changes in its ^1^H NMR spectrum were followed in DMSO (Figure S6), the solvent used to prepare the stock solutions for the bioassays. As the compound shows light sensitivity (*vide supra*), the measurements were performed under light-exclusion conditions. Over the 48 h monitoring period, the bidentate pyrithione ligand remained coordinated to the rhenium­(I) ion in the complex, while benzonitrile was completely replaced with a solvent molecule within 1 h (Scheme 2).

**Scheme 2:**
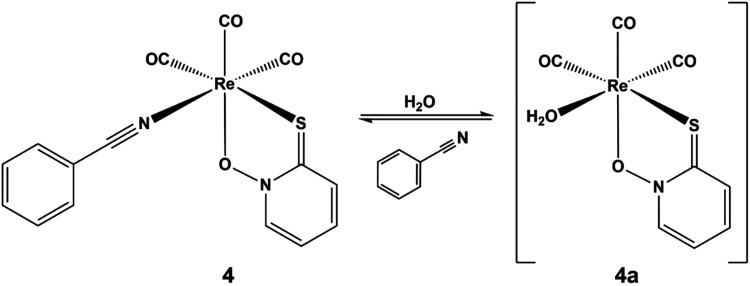
Transformation of Complex **4** to Hydrolysis Product **4a** in Aqueous Solution Observed in Solution Studies

The transformation processes of complex **4** were also studied in aqueous solution using UV–vis and ^1^H NMR spectroscopy as well as capillary zone electrophoresis (CZE). Measurements were performed in water (pH ∼ 6.8) and at pH 7.4 using buffers such as phosphate or 4-(2-hydroxyethyl)-1-piperazineethanesulfonic acid (HEPES). In the UV–vis spectra of the complex recorded over time in aqueous solution using samples kept in the absence of light (Figure 3), the observed spectral changes were minor, in contrast to the case in which the samples were exposed to light. After 80 h (in the presence of light), the absorbance bands within the 215–400 nm range significantly decreased, accompanied by the formation of a precipitate. The residual spectrum closely resembled that of free benzonitrile. These observations could be explained by the release of the monodentate coligand followed by a dimerization process of the complex, with the resulting product (compound **5**) exhibiting reduced aqueous solubility. Dimerization processes were also reported for similar complexes of 8-hydroxyquinoline ligands, in which the quinolinato O^–^ group bridges two rhenium centers.^46,48,49^

**Figure 3:**
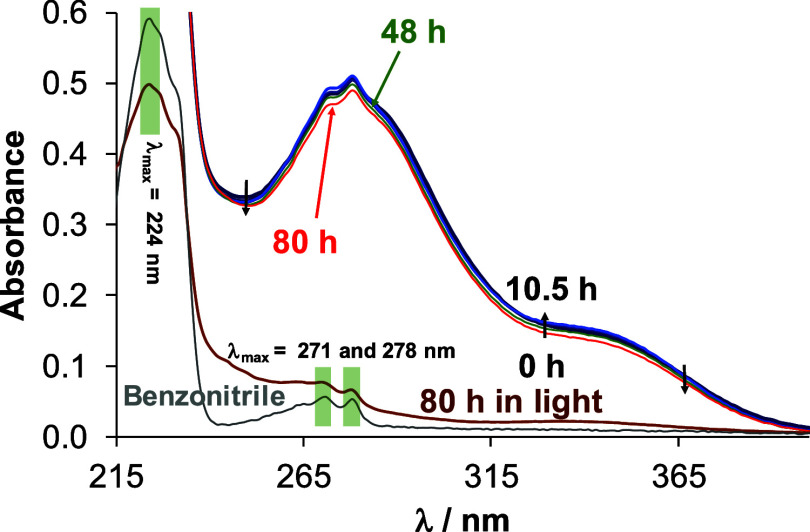
UV–vis spectra of complex **4** in water followed over time under light-exclusion conditions (0-80 h, colored lines). The spectrum of the light-exposed sample was also recorded after 80 h (brown line) as well as the spectrum of benzonitrile (gray line) at the same concentration as in the complex {*c*_complex_ = 50 μM; pH = 6.8; l = 1 cm; *T* = 25.0 °C}.

^1^H NMR spectra of samples kept in the dark also showed complete release of the benzonitrile ligand within 1 h at both pH 6.8 in water and pH 7.4 (in the presence of phosphate or HEPES buffer) (Figure S7). However, dissociation of the pyrithione ligand did not occur within 48 h. On the other hand, when the sample was exposed to light, only the peaks of benzonitrile were detected in the ^1^H NMR spectra along with formation of the precipitate. Therefore, the proposed formation of the dimeric species appears to be light-induced. Analysis of the electropherogram of the complex kept under light-exclusion conditions and the UV–vis spectra recorded at several migration times (Figure S8) also confirmed the presence of at least two species (complex [Re­(CO)_3_(pth)­(H_2_O)] (**4a**) and benzonitrile) in solution. Overall, based on the UV–vis, ^1^H NMR, and CZE results, it could be concluded that the dissociation of complex **4** to the aqua complex **4a** and the monodentate coligand occurs in a fast process upon its dissolution in water.

The behavior of complex **4** was also studied in Eagle’s Minimum Essential Medium (EMEM), and it was found that the changes in its UV–vis spectra over the 70 h time period were significant (Figure 4a), probably due to the coordination of an EMEM component replacing the monodentate ligand (the original benzonitrile to aqua). The appearance of a new set of peaks in the ^1^H NMR spectra (Figure 4b) confirmed coordination of an EMEM component without displacement of the pyrithione ligand. An attempt was made to test the behavior of complex **4** in blood serum, but precipitation at a concentration of 40 μM hindered the measurements.

**Figure 4:**
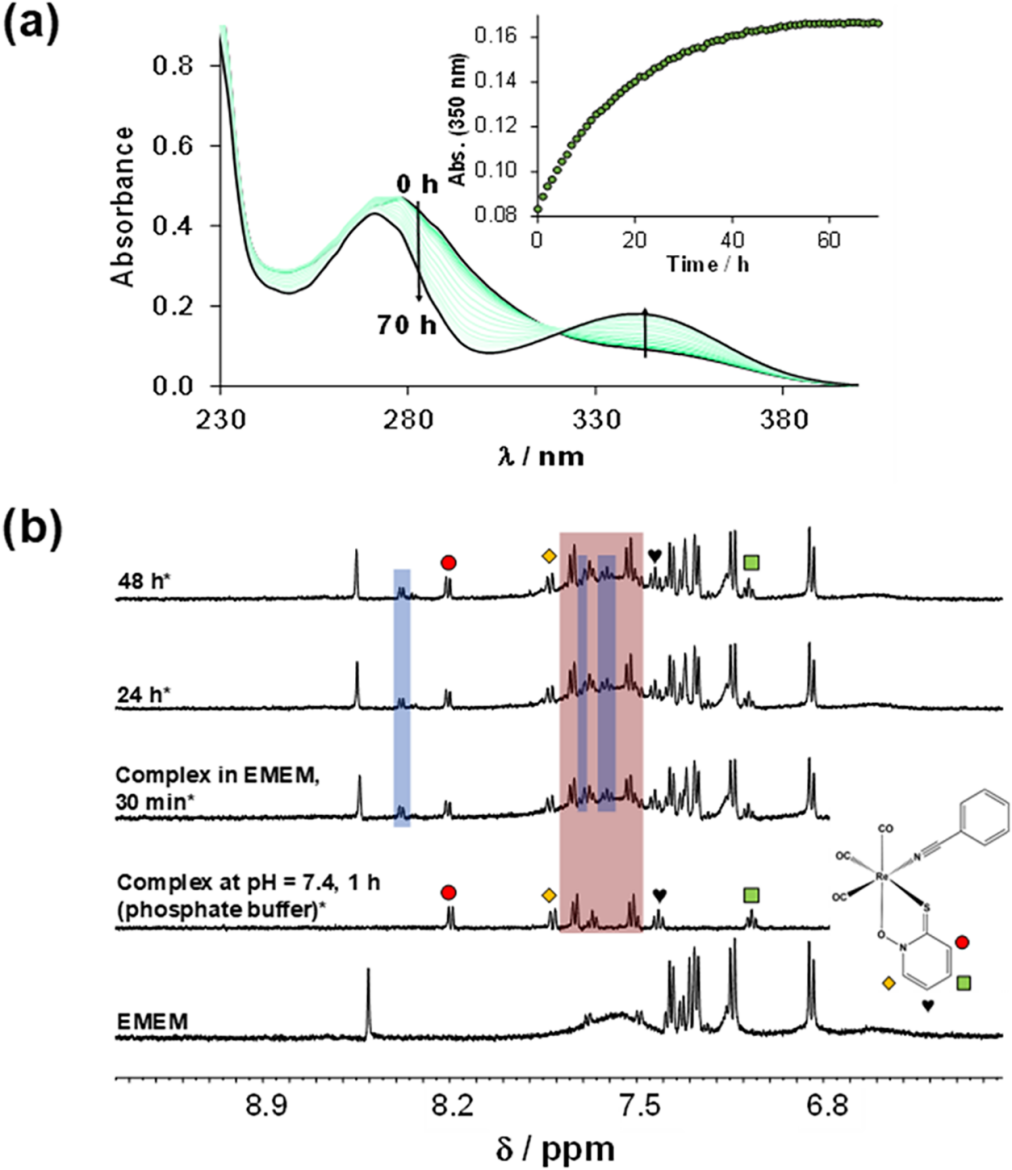
(a) UV–vis spectra of complex **4** in EMEM over time. Inset figure shows the absorbance values at 350 nm (green circle solid) plotted against time {*c*_complex_ = 50 μM; pH = 7.4; l = 1 cm; *T* = 25.0 °C}. (b) ^1^H NMR spectra of complex **4** in EMEM over time. Spectra of EMEM and the complex in phosphate buffer at pH 7.4 are also shown for comparison. Peaks of free benzonitrile are highlighted by a red rectangle. The new set of peaks is highlighted by blue rectangles. Samples (indicated with * symbol) were kept under light-exclusion conditions {*c*_complex_ = 250 μM; pH = 7.4; 10% (*v*/*v*) D_2_O/H_2_O; *T* = 25.0 °C}.

The solution speciation of complex **4** was further investigated in the pH range of 3–12 using UV–vis spectrophotometry. No spectral changes were observed between pH 3 and 8, indicating that the complex, most likely in the aqua form [Re­(CO)_3_(pth)­(H_2_O)] (**4a**), remains intact. However, at pH > ∼8, the spectrum changes considerably (Figure 5).

**Figure 5:**
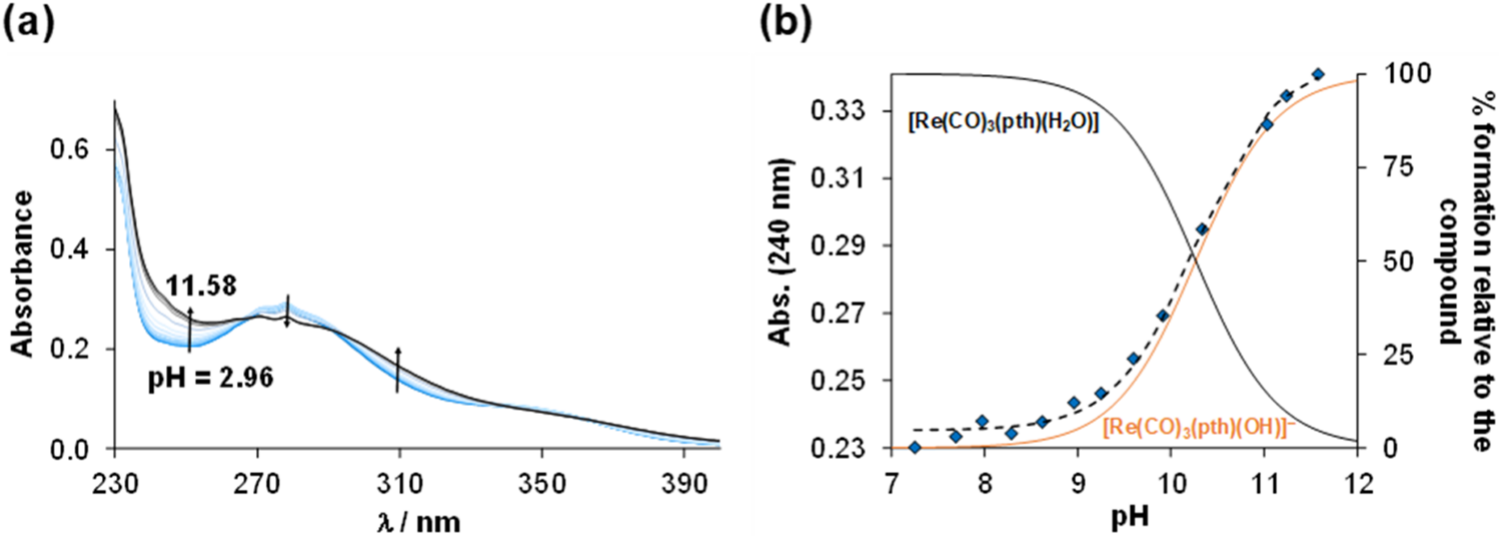
(a) UV–vis spectra of compound **4** at increasing pH values (2.96 → 11.58) under light-exclusion conditions. (b) Absorbance values at 240 nm (blue diamond solid) plotted against pH along with the fitted (dashed) line along with concentration distribution curves (black and orange solid lines) at pH > 7, where the deprotonation of the aqua complex **4a** takes place {*c*_complex_ = 30 μM; *I* = 0.1 M KCl; l = 1 cm; *T* = 25.0 °C}.

The isosbestic point indicates a single equilibrium process, which is most likely deprotonation of the coordinated aqua ligand in complex **4a**. This process was studied in our previous work for similar *fac*-[Re­(CO)_3_] complexes formed with (*N*,*N*)-bearing ligands, and the calculated p*K*_a_ (H_2_O) is around 8.^50^ Paira, Schutte-Smith, Visser et al. also reported p*K*_a_ values of rhenium-tricarbonyl ([Re­(CO)_3_(*L*,*L*)­(H_2_O)]), where (*L*,*L*) = (*N*,*N*) or (*O*,*O*), and rhenium-dicarbonyl ([Re­(CO)_2_(*N*,*N*)­(P)­(H_2_O)]^+^) complexes and the reported p*K*_a_ (H_2_O) values were between 8.3 and 9.3, indicating that deprotonation of the aqua ligand occurs in the basic pH range.^51–54^ Based on the changes in the spectra of complex **4a**, the proton dissociation constants were calculated: p*K*_a_ = 10.27 ± 0.01 (0.1 M KCl) and 10.15 ± 0.01 (0.1 M KNO_3_). Similar p*K*_a_ values in the presence and absence of the chloride ions indicate a low chloride affinity of the aqua complex. Based on these values, the fraction of [Re­(CO)_3_(pth)­(H_2_O)] is 100% at pH = 7.4. The analogous half-sandwich complex [Ru­(cym)­(pth)­(H_2_O)]^+^ was also characterized by high solution stability, and a similarly high p*K*_a_ value (10.34 (0.2 M KCl)).^55^ The weak chloride ion binding capacity was also confirmed when a large excess of chloride ions (∼7300) were added to the complex, and no changes were observed in the UV–vis spectra even after a 24 h waiting time (Figure S9).

The lipophilicity of complex **4** was characterized at different chloride ion concentrations (*c*_Cl_^–^ = 4, 24, and 100 mM, which are relevant for different biofluids such as the nucleus, cytosol, and serum, respectively, and in the absence of chloride ions). Distribution coefficients (*D*_7.4_) were determined using *n*-octanol/buffered aqueous solution (10 mM HEPES, pH = 7.4) partitioning (Table 2).

**Table 2:** Lipophilicity (at Different Chloride Ion Concentrations) of Complex **4**, Expressed as log* D*_7.4_, Measured via *n*-Octanol/Buffered Aqueous Solution Partitioning at pH = 7.4 (HEPES Buffer) along with Their Aqueous Solubility (*S*_7.4_) {*T* = 25.0 °C}^a^

	*c*(Cl^–^)/mM				
log* D*7.4	0	4	24	100	*S*_7.4_/μM
**4**	+1.61 ± 0.18	+1.67 ± 0.14	+1.71 ± 0.13	+1.71 ± 0.14	350 ± 10

a It is notable that the benzonitrile ligand is released from complex 4 upon dissolution.

Complex **4** exhibits moderate lipophilicity and the log *D*_7.4_ values show minimal variation with chloride ion concentration, confirming the low chloride ion affinity of the complex. The solubility of complex **4** was determined in water (at pH = 7.4, *I* = 0.1 M KCl), revealing a moderate aqueous solubility (Table 2).

### Interaction of Complex 4 with Human Serum Albumin

HSA is considered a key biomolecule in terms of the pharmacokinetic properties of a drug, as this abundant protein plays an important role in the transport and distribution of exogenous (*e.g*. drug molecules) and endogenous molecules. Furthermore, the binding of anticancer drugs to HSA has also attracted interest due to the enhanced permeability and retention effect.^56^ At first, the binding kinetics of complex **4** with HSA was followed by UV–vis spectrophotometry at a protein-to-complex ratio of 1:2 (Figure S10).

The UV–vis spectrum of complex **4** (mainly in the 300–400 nm range) changes upon interaction with HSA, most probably due to the changes in the coordination sphere where the aqua ligand may be replaced by a protein donor atom of an amino acid side chain. Notably, for the half-sandwich organometallic Ru­(II) and Rh­(III) complexes, the coordination of surface-exposed His side chain residues (through the His-imidazole nitrogen donor atom) was suggested, which can effectively replace the chlorido or aqua coligand, and for the studied *fac*-tricarbonylrhenium­(I) complex this type of binding is also possible.^57–59^ A similar binding pattern is also possible for these Re­(I) complexes. Therefore, the reaction of complex **4** was monitored with *N*-methylimidazole (MIM), which is used as a simplified histidine binding model (Figure S10).^57–59^ The spectral changes, together with the time required to reach equilibrium, closely resemble those observed with serum protein, suggesting a similar coordination sphere around the metal center, whether the model compound coordinates or the protein is bound to the complex. As an additional simplified protein binding model, acetylcysteine (Ac-Cys) was also used in the same way. However, no spectral changes could be observed over the 1 h period (Figure S11), suggesting that binding of HSA via a histidine nitrogen is the most likely.

To characterize the binding strength of the complex to HSA, the UV–vis spectrum of the complex was recorded in the presence of different amounts of the protein (Figure 6). The gradual spectral changes leveled off at about 0.5 equiv of the protein, indicating at least two binding sites. However, from these spectral changes, no formation constants could be calculated due to the overlapping processes. The ultrafiltration studies revealed a relatively strong binding affinity toward HSA, as only ∼10% of the complex could be detected as unbound species in the low molecular mass fraction at 1:2 protein-to-complex ratio (Figure S12).

**Figure 6:**
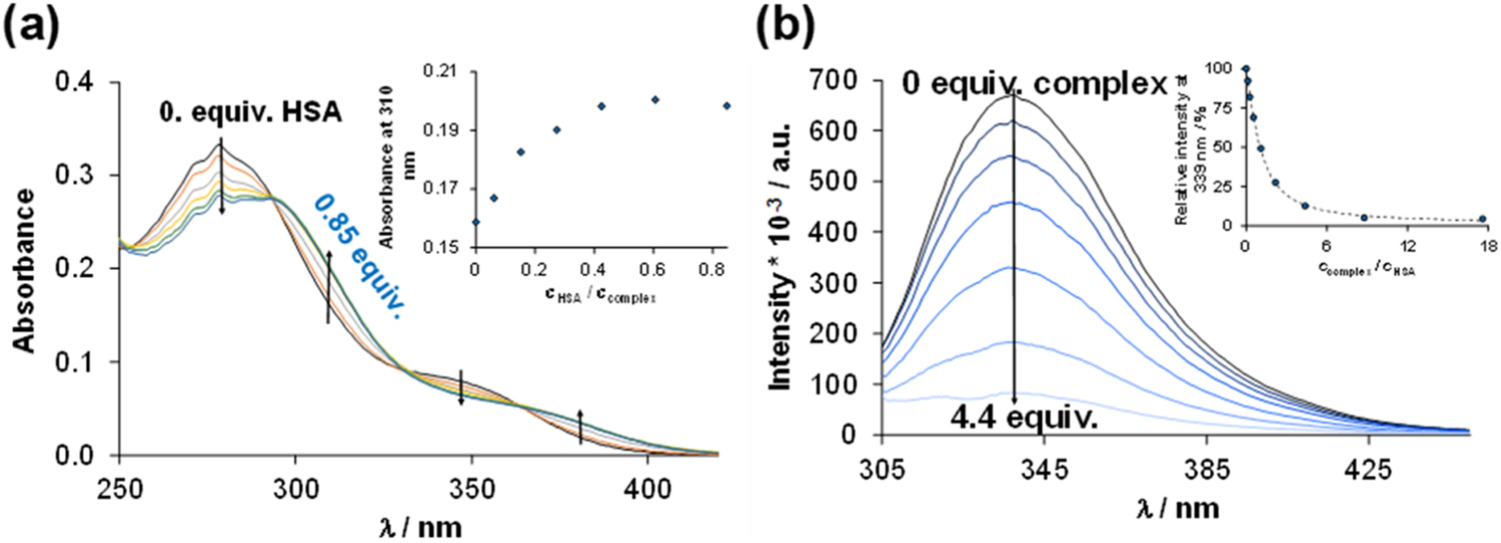
(a) UV–vis spectra of complex **4** in the presence of various equivalents of HSA under light-exclusion conditions. Inset figure shows the absorbance values (310 nm) as a function of *c*_HSA_/*c*_complex_ ratio {*c*_complex_ = 40 μM; *c*_HSA_= 0–34 μM; pH = 7.4 (20 mM phosphate buffer); l = 1 cm; *T* = 25.0 °C}. (b) Fluorescence emission spectra of HSA in the absence and presence of various equivalents of complex 4. Inset figure shows experimental (blue circle solid) and calculated (dashed line) relative emission intensities. Samples were kept under light-exclusion conditions {*c*_HSA_ = 1 μM; *c*_complex_ = 0–18 μM; λ_EX_ = 295 nm; pH = 7.4 (PBS buffer); *T* = 25 °C; l = 1 cm}. Note: The measurement was carried out as a titration of the complex with HSA.

The interaction of complex **4** with HSA was further investigated by using spectrofluorometry using the tryptophan quenching assay. HSA contains only one tryptophan residue (Trp214), which is located in a hydrophobic pocket in subdomain IIA of the protein. When a compound binds to HSA, it can affect the local environment of Trp214, leading to a decrease in its fluorescence intensity. The emission spectrum series of HSA with different equivalents of complex **4** (Figure 6) showed a high degree of quenching (∼85%) at low equivalents (∼4) of the complex. Based on the spectral changes, log *K*_Q_ = 6.3 ± 0.1 quenching constant was determined, indicating a strong interaction. A somewhat lower quenching constant was reported for the similar [Ru­(cym)­(pth)­Cl] complex (log* K*_Q_ = 5.81).^55^

### Evaluation of Pharmacological Activity of Complex 4

Organorhenium­(I) tricarbonyl complexes are widely investigated for their antitumor activity,^1,5,60^ and some complexes were reported to exhibit strong antiviral properties, including activity against SARS-CoV-2.^25,61^ This type of complex contains a bidentate ligand often with a (*N*,*N*) donor atom set. Pyrithione and its derivatives bearing an (*O*,*S*) donor set are also strong chelators for transition-metal ions such as Zn^2+^, VO^2+^, and Ru^2+^, and the formed complexes showed wide-range pharmacological activity including activity against SARS-CoV-2, antitumor activity, and antidiabetic activity.^33,62–65^ Compounds with dual or multiple therapeutic effects are of potential interest, particularly those that demonstrate synergy between anticancer, antibacterial, and antiviral properties.^66,67^

Here, complex **4** was tested for its anticancer, antibacterial, and antiviral activities under light-exclusion conditions. Its cytotoxicity was determined in the human HeLa cervical cancer cell line, the drug-sensitive Colo205 and its resistant counterpart, the ABCB1 expressing Colo320 colon adenocarcinoma cancer cell lines, using the colorimetric 3-(4,5-dimethylthiazol-2-yl)-2,5-diphenyltetrazolium bromide (MTT) test. To investigate the selectivity toward cancer cells, the noncancerous fibroblast CCD-19Lu cell line was included. The determined IC_50_ values are listed in Table 3. Complex **4** exhibited potent and similar cytotoxicity in the low micromolar concentration (IC_50_ = 8–10 μM) range against the cancer cell lines, and even lower IC_50_ was obtained for the noncancerous cell line, revealing no selectivity.

**Table 3:** *In Vitro* Cytotoxicity (Expressed as IC_50_ Values in μM) and Antibacterial Activity (Expressed as MIC: Minimum Inhibitory Concentration in μM) of Complex **4** and Benzonitrile^a^

	IC_50_/μM				MIC/μM		
	Colo205	Colo320	HeLa	CCD-19Lu	*S. aureus* ATCC 25923	*S. aureus* MRSA ATCC 43300	*E. coli* ATCC 25922
**4**	9.22 ± 0.53	10.48 ± 0.85	7.96 ± 0.98	5.47 ± 0.17	6.25	12.5	50
benzonitrile	>100	–	–	–	>100	>100	>100
doxorubicin	0.584 ± 0.055	3.13 ± 0.29	0.328 ± 0.049	0.244 ± 0.026	–	–	–

a Drug-sensitive (Colo205), resistant (Colo320) human colonic adenocarcinoma, cervical HeLa cancer, and noncancerous human fibroblast (CCD-19Lu) cell lines (72 h Incubation Time), and gram-positive *S. aureus* and gram-negative *E. coli* bacterial strains were used. Doxorubicin was used as the positive control.

The antibacterial effect of the complex was tested against the Gram-negative strain *Escherichia coli* (*E. coli*) ATCC 25922, the Gram-positive reference strain *Staphylococcus aureus* (*S. aureus*) ATCC 25923, and the methicillin-resistant *S. aureus* MRSA ATCC 43300 strain. MRSA strains are responsible for several difficult-to-treat hospital-acquired infections.^68^ The compound also showed considerable activity against the Gram-positive and Gram-negative strains investigated as the minimum inhibitory concentration (MIC) was obtained between MIC = 6.25 and 50 μM (Table 3), and it was the most active against the reference *S. aureus* bacterial strain. Benzonitrile was also included in the assays, and no significant anticancer or antibacterial activity was observed, which is in concurrence with the literature-reported very low cytotoxic effect.^69,70^

The complex was further tested against *H. simplex* virus 2 (HSV-2), which is a double-stranded DNA virus that infects mammals, including humans.^71^ To host the growing viruses, Vero cells (originally isolated from kidney epithelial cells from an African green monkey) were used, and MTT assay was performed on Vero cells to test the cytotoxicity, and the maximum nontoxic concentration was found to be 6.25 μM for the complex (Figure S13). Next, Vero cells were infected with HSV-2 at a multiplicity of infection (MOI) of 0.1. Subsequently, the cells were treated with the complex at different concentrations for 24 h. Afterward, the cells were lysed, and the antiviral efficacy of the complex was assessed by measuring the reduction in viral yield compared with untreated Vero cells. The growth of HSV-2 was monitored using a direct quantitative polymerase chain reaction (qPCR) analysis. Higher cycle threshold (Ct) values indicated that more PCR cycles were needed to detect the target nucleic acid, which signified stronger antiviral activity. The complex was capable of inhibiting the growth of the virus and showed stronger antiviral effect on HSV-2 virus compared to *fac*-[Re­(CO)_3_(bpy)­Cl] reference complex, which has been reported as an effective antiviral agent against SARS-CoV-2, as significantly lover Ct values (−17.7 to −14.1) in the concentration range 6.25–0.20 μM were detected for complex **4** compared to the reference complex (Figure 7).^61,72^

**Figure 7:**
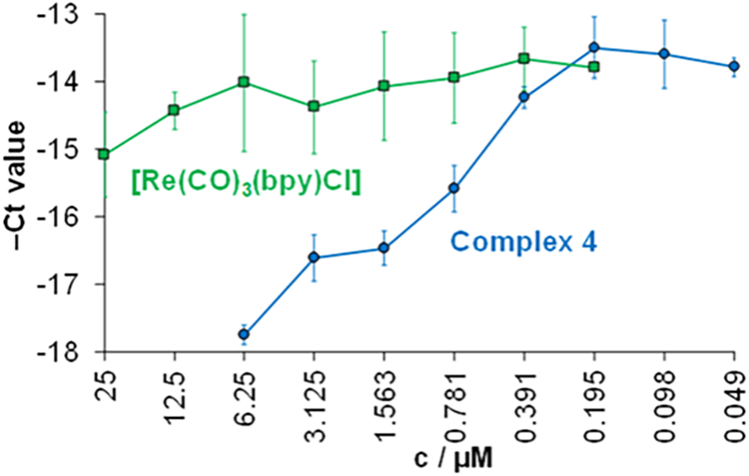
Antiviral activity of studied complex **4** against HSV-2 at different concentrations. The HSV-2 DNA concentration was measured by direct qPCR. Data represent the average – Ct values ± standard deviations. As a comparison, the antiviral activity of *fac*-[Re­(CO)_3_(bpy)­Cl] is represented, and data were taken from ref 50 {treatment of the Vero cells: 24 h; *n* = 3}. Reproduced from ref 50. Copyright 2024 American Chemical Society.

Since metal complexes can influence the qPCR reaction by interacting with the polymerase enzyme,^73^ the direct impact of the complexes on the DNA polymerase used in qPCR was also tested to ensure the reliability of the antiviral assay. Similar Ct values observed for the treated and untreated samples (complex: 17.01 ± 0.48; untreated: 16.67 ± 0.58) and the relatively low dCt value (0.34) indicate that there was neither stimulation nor inhibition during qPCR. For comparison, the [Ru­(cym)­(pth)­Cl] complex reported in our previous work showed comparable cytotoxicity as the present complex.^55^ However, the former complex possesses considerably weaker antibacterial effect (MIC = 50 μM only on *S. aureus* strain) than complex **4** and did not inhibit the growth of the HSV-2 virus.

### Mechanistic Insight into the Pharmacological Activity of the Complex

#### Evaluation of Cysteine Cathepsin Inhibition

Previously, we demonstrated that metal complexes can impair processes of tumor progression by inhibition of lysosomal cysteine peptidase cathepsin B.^35,36^ Additionally, mechanistic studies have shown that specifically rhenium compounds can be designed as active site-directed inhibitors of cathepsin B.^40^ Therefore, to get the mechanistic insight into the pharmacological activity of the complex here, we evaluated the effect of complex **4** for its inhibition of cysteine cathepsins. First, we used an initial enzyme kinetics assay to determine the relative inhibition of cathepsins B, V, and L by complex **4**. In addition to complex **4**, compounds [Re­(CO)_5_Cl], pthH, zinc pyrithione, and [Ru­(cym)­(pth)­Cl] were used as reference compounds (see the Supporting information (SI)). [Re­(CO)_5_Cl] and pyrithione are precursors for the preparation of complex **4** (Table 4), and zinc pyrithione and [Ru­(cym)­(pth)­Cl] were used in our previous studies and were tested for comparison (Table S2).

**Table 4:** Relative Inhibition of Cathepsins B, L, and V in % as Determined at a Concentration of 50 μM

	cathepsin B		cathepsin L	cathepsin V
	endopeptidase	exopeptidase		
**4**	63.6 ± 0.8	37.5 ± 2.1	5.0 ± 4.8	9.1 ± 1.0
[Re(CO)_5_Cl]	6.7 ± 0.7	12.2 ± 3.0	1.6 ± 6.5	4.6 ± 0.3
pthH	4.3 ± 2.3	n.i.^a^	0.5 ± 1.9	2.3 ± 3.2

a No inhibition.

Relative inhibition is expressed as a percentage of the decrease in reaction velocity in the presence of an inhibitor, in comparison to the reaction velocity in the absence of an inhibitor. For that purpose, the specific fluorogenic substrates Z-Arg-Arg-7-amido-4-methylcoumarin (Z-Arg-Arg-AMC) and 2-aminobenzoyl (Abz)-Gly-Ile-Val-Arg-Ala-Lys-(Dnp)-OH were used for cathepsin B endopeptidase and exopeptidase activities, respectively, and Z-Phe-Arg-AMC was used for cathepsin L and V activities. Data showed that relative inhibition, greater that 20%, approximate value indicating significant effect on enzyme activity, was observed only for the inhibition of cathepsin B endo- and exopeptidase activities by complex **4** (Table 4). Therefore, complex **4** was further characterized by determining the constants of inhibition for the activity of cathepsin B endopeptidase and exopeptidase (Table 5). In contrast to majority of cysteine cathepsins, cathepsin B possesses both endo- and exopeptidase activities due to the presence of the extra structural element termed the occluding loop that is pH-dependent and regulates the access of substrates to the active site of the enzyme.^74–79^ At lower pH, the occluding loop is attracted to the body of the enzyme by preventing the access of larger substrates to the active site and the enzyme cleaves substrates as exopeptidase. On the other hand, at higher pH, the salt bridges between the occluding loop and body of the enzyme are disrupted and a conformational change enables the access of larger substrates to the active site of the enzyme, resulting in the endopeptidase activity of the enzyme.^74–79^ The effect of complex **4** on both cathepsin B activities was therefore monitored. Complex **4** inhibited both cathepsin B activities in the micromolar range where a stronger effect with constant inhibition of 17.02 ± 1.59 μM was observed for inhibition of cathepsin B endopeptidase activity exhibiting an uncompetitive mode of inhibition, where the inhibitor predominantly inhibits cathepsin B activity by binding to the enzyme–substrate complex.

**Table 5:** Constants and Mechanisms of Inhibition of Cathepsin B Endopeptidase and Exopeptidase Activities for Complex **4**

		complex **4**	
	substrate	*K*_i_^a^ (μM)	*K*_i_′^b^ (μM)
endopeptidase activity	Z-RR-AMC		17.0 ± 1.6^c^
exopeptidase activity	Abz-GIVRAK(Dnp)-OH	165 ± 76^d^	67.0 ± 8.3^d^

a Dissociation constant for the dissociation of the enzyme–inhibitor complex.

b Dissociation constant for the dissociation of the enzyme–substrate–inhibitor complex.

c Uncompetitive inhibition.

d Mixed inhibition.

#### Effect of the Complex on the Degradation of ECM by Tumor Cells

Degradation of the extracellular matrix (ECM) is the process that enables tumor cells to migrate and invade into the surrounding tissue as well as to migrate to distant sites and form metastasis. Therefore, the process is essential for cancer progression.^80^ Proteolytic enzymes, including lysosomal cysteine cathepsin B, contribute to the degradation of ECM. ECM degradation by cathepsin B can occur extracellularly by secreted and membrane-associated enzymes or intracellularly within lysosomes following endocytosis of partially degraded components of ECM.^81–83^ In this study, we used fluorescently labeled collagen type IV, DQ-collagen IV, to investigate the effect of complex **4** on ECM degradation by MCF-10A neoT cells that were previously shown to degrade ECM both intracellularly and extracellularly.^83^ Collagen type IV is a major component of the ECM that can be tagged with fluorescein, and the proteolytic cleavage gives rise to bright green fluorescence. In addition to pthH and [Re­(CO)_5_Cl] that are precursors of complex **4**, known irreversible cathepsin inhibitors CA-074Me and CA-074 were included into the assay as controls (Figure 8). Data show that complex **4** significantly decreased both intracellular and extracellular DQ-collagen IV degradation by MCF-10A neoT cells. Intracellular DQ-collagen IV degradation was monitored by flow cytometry, while extracellular degradation was monitored spectrofluorometrically. Complex **4** reduced the intracellular DQ-collagen IV degradation for 83.3 ± 1.36% at 5 μM concentration (Figure 8a,b). Also, extracellular DQ-collagen IV degradation was reduced by complex **4** for 41.4 ± 5.18% (Figure 8c). In the concentrations that were used to monitor ECM degradation, complex **4** did not impair the viability of MCF-10A neoT cells as shown in Figure 9, where viability of the cells was monitored using MTS assay after treatment with inhibitors for 24 h.

**Figure 8:**
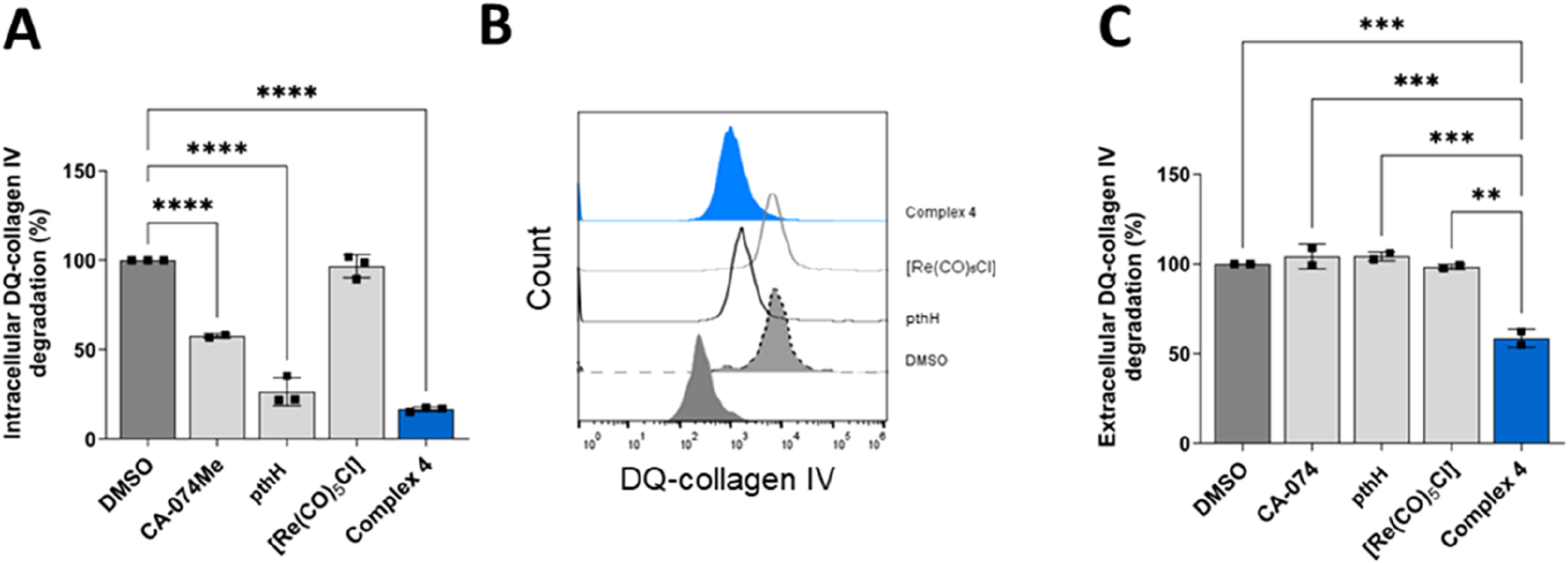
Complex **4** impairs intracellular and extracellular degradation of ECM by MCF-10A neoT cells. (A) Decrease of intracellular DQ-collagen IV degradation by MCF-10A neoT cells (6 × 10^4^) in the presence of DMSO, CA-074Me (50 μM) or complex **4**, ligand pthH, and [Re­(CO)_5_Cl] in concentrations of 5 and 50 μM (CA-074Me). Data are presented as mean ± SEM (*n* = 3) and experiments were performed in duplicates. (B) Intracellular degradation of DQ-collagen IV in the presence of DMSO or complex **4**, ligand pthH and [Re­(CO)_5_Cl] shown as change in the fluorescence intensity as measured by flow cytometer. (C) Extracellular degradation of DQ-collagen IV by MCF-10A neoT cells (5 × 10^4^) in the presence of DMSO, CA-074 (5 μM) or complex **4**, pthH, and [Re­(CO)_5_Cl] (5 μM). Extracellular degradation of DQ-collagen was analyzed by monitoring the fluorescence intensity of the extracellular degradation product using spectrofluorimetry. Data is presented as mean ± SEM (*n* = 2). *P < 0.05, **P < 0.01, ***P < 0.001, and ****P < 0.0001.

**Figure 9:**
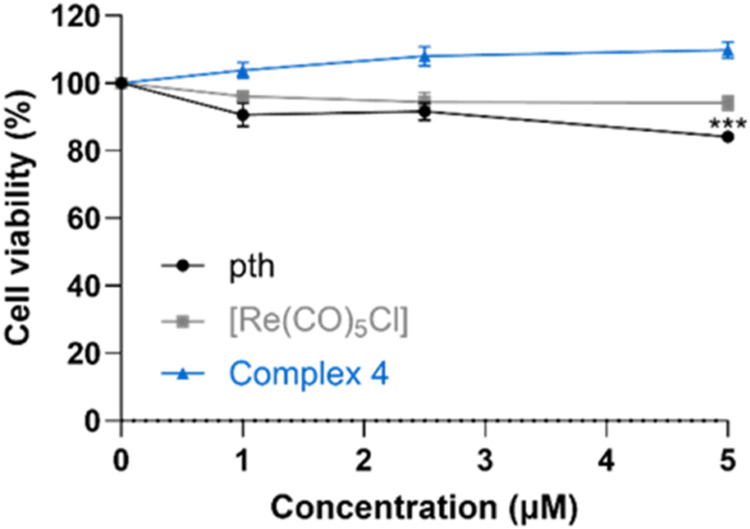
Effect of the compounds on MCF-10A neoT cell viability as determined by the MTS assay. MCF-10A neoT cells (6 × 10^4^) were treated with DMSO or compounds for 24 h. Data are presented as a percentage of viable cells from two independent experiments (mean ± SEM) in the presence of the inhibitor in comparison to DMSO, which was used as a control. The experiments were performed in quadruplicate. *P < 0.05, **P < 0.01, and ***P < 0.001.

### In Silico Studies

Computational studies were undertaken to try to explain the binding of complexes **4** to human cathepsin B. As the benzonitrile ligand of **4** was observed to undergo fast dissociation in aqueous solutions, we also modeled its aqua form **4a**. The V-shaped active site cleft of cathepsin B is located between left and right domains of the enzyme and contains the catalytic triad (nucleophilic Cys29, general base His199, and Asn220) that forms a hydrogen bond with His199 to help maintain the correct orientation of the active site.^74,84^ Additionally, the activity of cathepsin B is defined by the presence of an extra structural element termed the occluding loop that regulates the access of substrates to the active site cleft, as described above. Both **4** and **4a** were able to accommodate themselves within the active site cavity, 5.0 and 4.5 Å away from the catalytic Cys29, respectively (measured from *S*-Cys to Re atom) (Figure 10; A,B). The **4a**-human cathepsin B noncovalent complex was additionally subjected to molecular dynamics (MD) to evaluate the stability of the predicted pose over 100 ns. During the simulation, ligand **4a** moved away from catalytic Cys29 (up to 8.4 Å in the final frame) and remained relatively stable in the vicinity of Trp221 and His110 (Figure 10; C,D). Nonetheless, it still occludes the active site cavity, which could prevent the access of substrate and lead to enzyme inhibition. Additionally, as coordination (covalent binding) of **4a** to catalytic Cys29 is also possible, the dynamic stability of covalently bound **4a** was also evaluated by using MD (Figure 10; E,F). As expected, the bound complex interacted with the catalytic triad. In addition, the *N*-oxide moiety of the coordinated pyrithione ligand was involved in persistent hydrogen bonds with Gln23 and Trp22. All in all, *in silico* experiments demonstrated that binding of complexes **4** and **4a** to the human cathepsin B active site is possible and can lead to inhibition of enzymatic activity.

**Figure 10:**
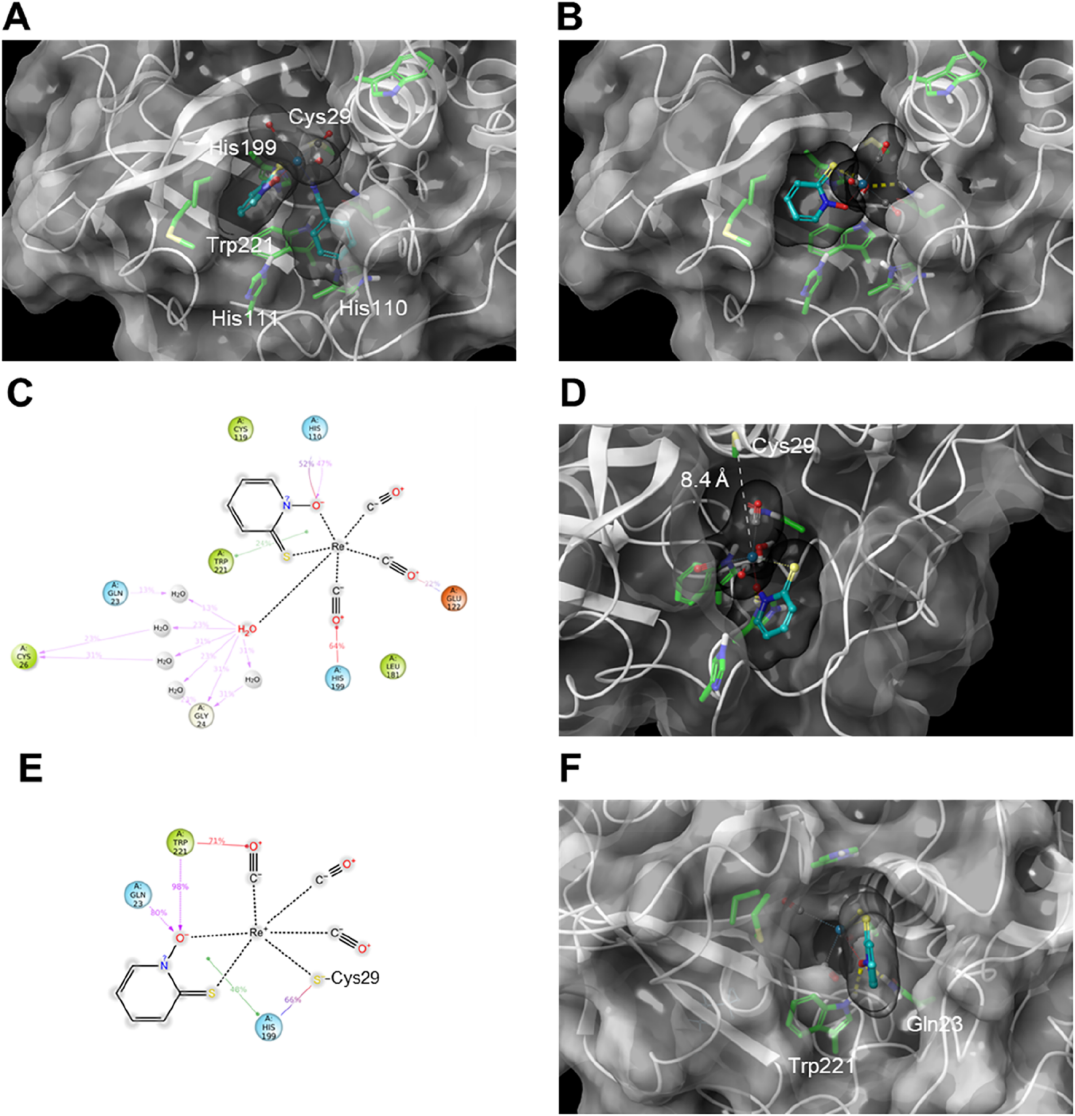
(A) Docked pose of complex **4** in the active site of human cathepsin B. The key amino acid residues are labeled and shown as green sticks, whereas the ligand is shown as azure balls and sticks. The semitransparent molecular surfaces of the protein and ligand are shown in white and black, respectively. (B) Docked pose of complex **4a** in the active site of human cathepsin B. (C) Ligand interaction diagram based on 100 ns MD simulation of complex **4a** in the human cathepsin B active site. Protein–ligand contacts and interactions that occur for more than 10% of the MD simulation time are shown: π–π interactions as green lines, cation−π interactions as red lines, and hydrogen bonds are shown in magenta. Gray circles denote solvent exposure. (D) Last frame of 100 ns MD simulation of complex **4a-**human cathepsin B. Distance between Re and *S*-Cys29 atoms is shown as a white dashed line. (E) Ligand interaction diagram based on 100 ns MD simulation of complex **4a** bound to human cathepsin B via coordination bond (the aqua ligand of **4a** was exchanged for the catalytic Cys29 thiolate). (F) Last frame of 100 ns MD simulation of complex **4a** bound to human cathepsin B via coordination. *N*-oxide moiety of the coordinated pyrithione ligand is involved in persistent hydrogen bonds with Gln23 and Trp221, shown as yellow dashed lines.

## Conclusions

A new *fac*-tricarbonylrhenium­(I) complex (**4**) with pyrithione ligand and benzonitrile coligand was synthesized. Detailed synthesis of the chemical reactivity of selected rhenium­(I) precursors with pyrithione and/or 2-mercaptopyridine in the absence or presence of acetonitrile or benzonitrile was presented together with a complete physicochemical characterization of complex **4**. We were able to identify and fully or partially characterize six different products that were obtained under different reaction conditions. Ultimately, only complex **4** showed sufficient chemical stability and aqueous solubility to be included in the biological screenings.

The stability of complex **4** was analyzed in DMSO and in various biologically relevant matrices. Due to the photosensitivity of the complex, investigations were carried out in the absence of light, and some measurements were also performed in the presence of light for comparison. During the time span of the experiment, the carbonyl and pyrithione ligands remained bound to rhenium, while the benzonitrile coligand was released from the complex and exchanged for a solvent molecule or a compound from the biological matrices. In addition, speciation in aqueous solution was studied over a wide pH range and it was found that complex **4** (in the form of the aqua complex following dissolution) is stable in the pH range of 3–12; however, above pH 8, the deprotonation of the coordinated aqua ligand most likely occurs. The complex has low chloride ion affinity, moderate lipophilicity, and solubility at pH = 7.4. Complex **4** interacts with human serum albumin, establishing coordinative binding mode. Among others, its strong binding affinity for the protein was confirmed by a high degree of fluorescence quenching.

Complex **4** showed strong cytotoxicity in the single-digit micromolar concentrations range (IC_50_ = 8–10 μM) on Colo205, Colo320, and HeLa human cancer cell lines. The antibacterial effect on Gram-positive *S. aureus* and *S. aureus* MRSA as well as Gram-negative *E. coli* showed noticeable activity (MIC = 6.25–50 μM). The antiviral activity was investigated with *H. simplex* virus type 2 (HSV-2) in Vero host cells, where the complex was able to significantly inhibit the growth of the virus.

Mechanistic insights into the pharmacological activity of the complex were investigated by assessing inhibition of cysteine cathepsin B, cathepsin L, and cathepsin V. Significant inhibition was observed for the endopeptidase and exopeptidase activities of cathepsin B. The complex was able to reduce the amount of intra- and exocellular degradation of the ECM, while the cells remained viable. Interaction of complex **4** and its hydrolyzed form **4a** with cathepsin B was also investigated by computational studies, which confirmed that both reversible and covalent binding of the complex in the active site of cathepsin B is possible.

This study represents an important first step in the systematic approach to the synthesis of rhenium carbonyl complexes with pyrithione and reveals the rich chemistry behind the system. Encouraging results of the biological assays have revealed the hidden potential of *fac*-tricarbonylrhenium­(I) complexes with pyrithione ligand.

## Materials and Methods

### Chemicals and General Information

All starting materials for the synthesis were purchased from commercial sources (Strem Chemicals, Merck) and used as received. Solvents for the reaction were dried over sodium sulfate and molecular sieves, while solvents for isolation of the compounds were used without further purification or drying.

All other solvents were of analytical grade and used without further purification. KOH, HEPES, HSA (A8763, essentially globulin free), D_2_O, 1-methylimidazole, acetylcysteine, EMEM media, doxorubicin, cisplatin, and human serum (from human male AB plasma) were Sigma-Aldrich/Merck (St. Louis, MO) products and used without further purification. DMSO, KCl, KNO_3_, HCl, HNO_3_, NaH_2_PO_4_, Na_2_HPO_4_, KH_2_PO_4_, and *n*-octanol were Molar Chemicals (Halásztelek, Hungary) products. DMSO-*d*_6_ was purchased from VWR Chemicals (Radnor, Pennsylvania).

For the characterization of the compounds, NMR spectroscopy was performed on a Bruker Avance Neo 600 spectrometer at room temperature. ^1^H NMR spectra were recorded at 600 MHz. Chemical shifts are referenced to residual peaks of the deuterated solvent MeOD-*d*_4_ at 3.31 ppm. Chemical shifts (δ) and coupling constants (*J*) are given in parts per million and Hz, respectively. All NMR data were processed using MestRe-Nova version 14.2.3. Infrared spectra were recorded on a PerkinElmer Spectrum Two with ATR. High-resolution mass spectra (HRMS) were recorded on an Agilent 6224 Accurate Mass TOF LC/MS instrument. Elemental analyses (CHN) were carried out on a PerkinElmer 2400 II instrument. UV/vis spectra were collected on a PerkinElmer Lambda 750 UV/vis/near-IR spectrophotometer. IR and UV–vis data were processed using Spectragryph.^85^

Single-crystal X-ray diffraction data were collected at 150 K on a SuperNova diffractometer with Atlas detector using CrysAlis software with monochromated Mo Kα (0.71073 Å).^86^ The initial structural models were solved with direct methods implemented in SHELXT using the *Olex*2 graphical user interface.^87^ A full-matrix least-squares refinement on *F* ^2^ magnitudes with anisotropic displacement parameters for all nonhydrogen atoms using *Olex*2 or *SHELXL*-2018/3 was performed.^87,88^ All nonhydrogen atoms were refined anisotropically, while hydrogen atoms were placed at calculated positions and treated as riding on their parent atoms. Details on the crystal data, data acquisition, and refinement are presented in Table S1. Mercury^89^ was used for the preparation of the figures. CCDC deposition numbers 2423360–2423365 contain the supplementary crystallographic data for this paper.

In this work, risk was controlled by following the standard laboratory safety rules. When handling chemicals, personal protecting equipment was utilized. None of the chemicals used in this work present a high danger or high risk when handling.

Additional synthesis details for other obtained products are provided in the SI materials.

### Synthesis of *fac*-[Re­(CO)_3_(pth)­(benzonitrile)] Complex (**4**)

Rhenium complex **4** was synthesized in three steps.^47,90^ In the first step, [Re­(CO)_5_Cl] was heated at 110 °C for 72 h in 12 mL of distilled water in a high-pressure tube. After the reaction was completed, all starting material reacted with water. The reaction mixture was transferred to a 50 mL flask and concentrated under reduced pressure. Sodium pyrithione (1 mol equiv) in 20 mL of MeOH was added, and the reaction mixture was stirred overnight protected from light. After 24 h, benzonitrile (4 mol equiv) was added and the reaction mixture was again stirred overnight, protected from light. After 24 h, the precipitate was collected with vacuum filtration and washed with 20 mL of hexane. The product was dried at room temperature in the dark.

**4**: Yield 77.3% (106.7 mg), yellow solid. ^1^H NMR (600 MHz, MeOD-*d*_4_) δ 8.32 (ddd, *J* = 6.8, 1.4, 0.6 Hz, 1H), 7.79 (ddd, *J* = 8.4, 1.7, 0.6 Hz, 1H), 7.75–7.71 (m, 2H), 7.71–7.66 (m, 1H), 7.58–7.52 (m, 2H), 7.35 (ddd, *J* = 8.4, 7.1, 1.4 Hz, 1H), 7.00 (td, *J* = 6.9, 1.8 Hz, 1H). IR selected bands (ATR): $\upsilon^{\prime }$ = 3113, 3036, 2267, 2009, 1949, 1880, 1595, 1542, 1414, 1266, 1235, 1139, 1087, 819, 755, 710, 684, 516, 478 cm^–1^. UV–vis (λ (nm) (ε (M^–1^cm^–1^)), *c* = 1 × 10^–5^ M, MeOH): (290, 357), (15,573, 3595.6). ESI-HRMS (acetonitrile) for [M-BN + 2CH_3_CN]^+^ found: 480.0027 (calcd: 480.0022). Elemental analysis for C_15_H_9_N_2_O_4_ReS calcd (%): C: 36.07; H: 1.82; N: 5.61; found (%): C: 36.15; H: 1.63; N: 5.11.

### Stock Solutions and Sample Preparation

Milli-Q water or DMSO was used for the preparation of stock and sample solutions. Stock solutions of complex were obtained by dissolving an exact amount in water or DMSO and their concentrations were calculated based on a weight-in-volume basis. MIM stock solution was made in water and its concentration was determined by pH-potentiometry.^91^ HSA stock solutions were prepared in modified phosphate-buffered saline (PBS′), which contains 12 mM Na_2_HPO_4_, 3 mM KH_2_PO_4_, 1.5 mM KCl, and 100.5 mM NaCl; the concentrations of K^+^, Na^+^, and Cl^–^ ions correspond to the human blood serum. Residual citrate content of HSA was removed by repeated ultrafiltration of the protein stock solution, and its concentration was calculated from its UV absorption: λ_280 nm_ (HSA) = 36,850 M^–1^ cm^–1^.^92^ Samples were prepared in PBS’ to study the interaction with HSA at 25 °C.

### UV–Visible Spectrophotometry

For the solution studies, an Agilent Cary 3500 spectrophotometer was utilized to obtain UV–vis spectra in the wavelength range of 200–1100 nm. The path length ( l ) was 1 cm in all cases. The concentrations of the complex were between 10 and 60 μM. For investigation of the effect of chloride ions, the samples contained 60 μM complex and 0–7300 equiv of Cl^–^. For HSA-containing samples, complex concentrations were 60 μM, and 0.5 equiv of biomolecule (or 6 equiv of MIM) was added for kinetic studies, respectively. Individual samples contained 0–34 μM HSA. The computer program HypSpec was used to obtain equilibrium constants.^91^

### Determination of Distribution Coefficients

The traditional shake-flask method was used to obtain the distribution coefficient of the complex in *n*-octanol/buffered aqueous solution (10 mM HEPES, pH = 7.4) using UV–vis spectrophotometry. The measurements and data evaluation were performed as in our previous work.^93^

### Determination of Aqueous Solubility

Thermodynamic solubility (*S*_7.4_) of complex **4** was assessed by measuring the saturation levels in water at pH = 7.4 (10 mM HEPES buffer) at 25.0 ± 0.1 °C. The concentration of the compound was determined by UV–vis spectrophotometry. For calibration, stock solutions of the compound were used with known concentrations dissolved in 100% DMSO, 75% DMSO, and 50% (*v*/*v*) DMSO/buffered aqueous solutions.

### Solution Studies Applying NMR Spectroscopy

A Bruker Avance III HD Ascend 500 Plus instrument (Billerica, MA) was used for solution NMR studies. Spectra were recorded with a WATERGATE water suppression pulse scheme in the presence of 10% (*v*/*v*) D_2_O in most cases. DSS internal standard was added to samples to obtain reference peaks.

### Spectrofluorometry

Fluorescence measurements were carried out on a Fluoromax (Horiba Jobin Yvon, Longjumeau, France) spectrofluorometer using a 1 cm × 1 cm quartz cuvette. Samples contained 1 μM HSA and the complex concentration was varied between 0 and 18 μM. The measurements were carried out as titration. The excitation wavelength was 295 for the Trp214 quenching measurements, and the determined quenching constant for HSA–complex species (1:1) was obtained using the computer program HypSpec.^91^ Calculations always were based on data obtained from at least two independent measurements. Self-absorbance and inner filter effect had to be considered,^94^ and corrections were made as was described in our former works.^95,96^

### Capillary Zone Electrophoresis

An Agilent 7100 capillary electrophoresis system equipped with a diode-array UV–vis detector (200–600 nm) was utilized to gain electropherograms. Fused silica capillaries of 48 cm total length with a 75 μm inner diameter were used (Agilent Technologies, Santa Clara, CA). The background electrolyte (BGE) was 10 mM HEPES buffer (pH = 7.4), in which the samples were also made. The conditioning process of new capillaries and daily preparation were performed as described formerly.^69^ In order to ensure the steady baseline, the capillary was flushed with BGE (2 min) before each run and was rinsed with NaOH (0.1 M; 1.5 min), H_2_O (1.5 min), and then with BGE (2 min) after each run. As postconditioning, the capillary was also flushed with BGE for 1 min. The sample tray and the capillary cassette were kept at 25 °C. The hydrodynamic injection was used at 100 mbar for a 5 s injection time. For separation, 25 kV voltage was applied, which produced *ca*. 12.5 μA current. The sample run time was set to 6 min. The computer program ChemStation (Agilent)^97^ was used to record electropherograms. Electropherograms and UV–vis spectra of the corresponding peaks were also collected.

### Membrane Ultrafiltration

Milli-pore, Amicon Ultra-0.5 membrane filters (10 kDa) were used to separate samples containing 25 μM HSA and 50 μM complex (1:2 biomolecule-to-complex ratio) into low and high molecular mass (LMM and HMM) fractions as described in our former work.^98^ UV–vis spectrophotometry was used to determine the concentration of the nonbound compounds in the LMM fractions by comparing to a reference sample without the protein.

### Cell Lines and Culture Conditions

Drug-sensitive Colo205 (ATCC-CCL-222) and ABCB1 (MDR1)-LRP expressing and doxorubicin-resistant Colo320/MDR-LRP (ATCC-CCL-220.1) human colonic adenocarcinoma cell lines were purchased from LGC Promochem (Teddington, U.K.). The cells were cultured in RPMI 1640 medium supplemented with 10% heat-inactivated fetal bovine serum, 2 mM l-glutamine, 1 mM Na-pyruvate, and 10 mM HEPES.

HeLa 229 cells (ATCC-CCL-2.1) were cultured in minimal essential medium (MEM) (Sigma, Steinheim, Germany) with Earle salts supplemented with 10% heat-inactivated fetal bovine serum (FBS) (Gibco; Thermo Fisher Scientific, Inc., Waltham, MA), 2 mM l-glutamine, 1× nonessential amino acids, 8 mM HEPES, 25 μg/mL gentamycin, and 1 μg/mL fungisone.

The CCD-19Lu (CCL-210) human normal fibroblast cell line was purchased from American Type Culture Collection (ATCC). CCD-19Lu cells were cultured in EMEM (Sigma-Aldrich, St Louis, MO) supplemented with a nonessential amino acid (NEAA) mixture (Sigma-Aldrich, St Louis, MO), a selection of vitamins, 10% heat-inactivated FBS, 2 mM l-glutamine (Sigma-Aldrich, St Louis, MO), 1 mM Na-pyruvate (Sigma-Aldrich, St Louis, MO), nystatin (Sigma-Aldrich, St Louis, MO), and gentamicin (Sigma-Aldrich, St Louis, MO).

MCF-10A neoT, a c-Ha-ras oncogene transfected human mammary epithelial cell line, was provided by Bonnie F. Sloane (Wayne State University, Detroit, MI). MCF-10A neoT cells were Dulbecco’s modified Eagle’s medium (DMEM)/Nutrient Mixture F-12 (1:1) medium with GlutaMAX (Gibco, Carlsbad, CA) supplemented with 5% fetal bovine serum (FBS, Gibco), 1 μg/mL insulin (Sigma-Aldrich), 0.5 μg/mL hydrocortisone (Sigma-Aldrich), 20 ng/mL epithelial growth factor (Milipore, Burlington, MA), and 1% penicillin–streptomycin (Gibco), corresponding to 100 U/mL penicillin and 100 μg/mL streptomycin.

The cells were incubated under 5% CO_2_, 95% air atmosphere at 37 °C. The MCF-10A neoT cell line was detached with or TripLE Select Enzyme (Gibco), while other cell lines were detached with Trypsin-versene (EDTA) solution for 5 min at 37 °C.

### *In Vitro* Cytotoxicity Assay

#### MTT Assay

The tested compound was dissolved in DMSO to prepare a 5 mM stock solution, which was diluted in complete culture medium, to study the effect of compounds on cell growth of human colonic adenocarcinoma cell lines. Doxorubicin was used as the positive control. The cells were treated with a trypsin-versene (EDTA) solution. Cells were adjusted to a density of 1 × 10^4^ cells in 100 μL of the appropriate culture medium and were added to each well, with the exception of the medium control wells. Except for the semiadherent Colo205 and Colo320 cell lines, the other adherent cell lines were seeded 24 h prior to the assay. Then, stock solutions were diluted in the appropriate culture medium, and 2-fold serial dilutions of compounds were prepared in 100 μL of the medium, horizontally. The final volume of the wells containing compounds and cells was 200 μL. The plates were incubated at 37 °C for 72 h; at the end of the incubation period, 20 μL of MTT solution (from a stock solution of 5 mg/mL) was added to each well. After incubation at 37 °C for 4 h, 100 μL of SDS solution (10% in 0.01 M HCI) was added to each well, and the plates were further incubated at 37 °C overnight. Cell growth was determined by measuring the optical density (OD) at 540/630 nm with a Multiscan EX ELISA reader (Thermo Labsystems, Cheshire, WA). Inhibition of the cell growth (expressed as IC_50_: inhibitory concentration that reduces by 50% the growth of the cells exposed to the tested compounds) was determined from the sigmoid curve where 100 – ((OD_sample_ – OD_medium control_)/(OD_cell control_ – OD_medium control_)) × 100 values were plotted against the logarithm of compound concentrations. Curves were fitted by GraphPad Prism software (2021, Graphpad Software, San Diego, CA)^99^ using the sigmoidal dose–response model (comparing variable and fixed slopes). The IC_50_ values were obtained from at least 3 independent experiments.

#### MTS Assay

The effect of complex **4**, [Re­(CO)_5_Cl], and pyrithione on the viability of MCF-10A neoT was evaluated using the MTS [3-(4,5-dimethylthiazol-2-yl)-5-(3-carboxymethoxyphenyl)-2-(4-sulfophenyl)-2*H*-tetrazolium] colorimetric assay (CellTiter 96 Aqueous One Solution Cell Proliferation Assay, Promega, Madison, WI). The cells were seeded at 6 × 10^4^ cells/well into a 96-well microplate and incubated overnight for attachment. The cells were then treated with the inhibitor (at 1, 2.5, or 5 μM) or DMSO (0.1%) in 100 μL for 24 h. After incubation, 10 μL of the reagent MTS was added to the wells, and the absorbance of formazan was measured at 492 nm on a Tecan Infinite M1000 instrument (Mannedorf, Switzerland). Cell viability (%) was expressed as the ratio between absorbance in the presence of the compounds and that in the presence of DMSO. All assays were performed in quadruplicate and repeated twice.

### DQ-Collagen Degradation Assay

To determine the effect of rhenium complex **4** on the degradation of the ECM, we monitored the degradation of DQ-collagen type IV. Intracellular degradation of DQ-collagen IV was monitored by flow cytometry, and extracellular degradation was monitored with spectrofluorimetry.

To monitor intracellular degradation of DQ-collagen type IV, MCF-10A neoT cells were seeded at 6 × 10^4^ cells/well into a 24-well plate and incubated overnight at 37 °C to adhere. The cells were then treated with complex **4**, [Re­(CO)_5_Cl] and pyrithione (all 5 μM), selective cathepsin B inhibitor CA-074Me (50 μM) or DMSO (0.5%) in 500 μL of serum-free medium (SFM) for 2 h at 37 °C. After 2 h of incubation, DQ-collagen IV (5 μg/mL; Thermo Fischer, Rockford, IL) was added, and the cells were incubated for an additional 2 h at 37 °C. The cells were detached and washed with phosphate-buffered saline (PBS). Propidium iodide (10 μg/mL; BD Bioscience) was added to exclude dead cells, and thus green fluorescence resulting from the degradation of DQ-collagen IV was monitored only for viable cells. The measurement was performed using the Attune NxT flow cytometer (Thermo Fischer). The assay was performed in duplicates and repeated three times.

To monitor extracellular degradation of DQ-collagen type IV, MCF-10A neoT cells were seeded at 5 × 10^4^ cells/well into a 96-well microplate and allowed to adhere overnight at 37 °C. The next day, the cells were treated with complex **4**, [Re­(CO)_5_Cl] and pyrithione (all 5 μM), selective cathepsin B inhibitor CA-074 (5 μM) or DMSO (0.5%), and DQ-collagen IV (10 μg/mL) in 100 μL of PBS at 37 °C for 6 h. After incubation, the reaction mixture (80 μL) was transferred to empty wells of a black 96-well microplate, where fluorescence intensity was continuously monitored for 2 h at 515 ± 5 nm with excitation at 495 ± 5 nm on a Tecan Infinite M1000 microplate reader. Inhibition of extracellular DQ-collagen IV degradation was expressed as the ratio between the average 2 h fluorescence obtained in the presence of the compounds and DMSO. The assay was performed in six parallels and repeated two times.

### Antibacterial Activity Assay

The following bacterial strains were used in our experiments: Gram-positive *S. aureus* American Type Culture Collection (ATCC) 25923 as the methicillin-susceptible reference bacterial strain; methicillin-resistant *S. aureus* ATCC 43300 (MRSA) strain; *E. coli* ATCC 25922 as a Gram-negative bacterial strain.

MIC values of the complexes were determined in 96-well plates based on the Clinical and Laboratory Standard Institute guidelines (CLSI guidelines).^100^ The stock solutions of the compound (dissolved in DMSO at 5 mM concentration) were diluted in 100 μL of Mueller Hinton Broth. Then, 10^–4^ dilution of an overnight bacterial culture in 100 μL of medium was added to each well, with the exception of the medium control wells. The plates were further incubated at 37 °C for 18 h; at the end of the incubation period, the MIC values of the tested compounds were determined by visual inspection.

### Antiviral Activity Assay

#### Cultivation and Quantification of Herpes Simplex Virus Type 2

HSV-2 strain was (gift from Dr. Ilona Mucsi, University of Szeged, Szeged, Hungary) grown in Vero cells and the infectivity was determined by using the plaque titration method.^101^ The virus titer was expressed as plaque forming units (PFU).^102^

#### Culture of Vero Cells and MTT Assay

Vero cells (ATCC-CCL-81) were placed into a 96-well plate (Sarstedt, Nümbrecht, Germany) at a density of 4 × 10^6^ cells/plate. The cells at a density of 4 × 10^4^ cells per well were in 100 μL of minimal essential medium (MEM) (Sigma) with Earle salts supplemented with 25 μg/mL gentamycin, 10% heat-inactivated fetal bovine serum (FBS) (Gibco; Germany), 8 mM HEPES, 2 mM l-glutamine, 1× nonessential amino acids, and 1 μg/mL fungisone. The cells were incubated for 60 min at room temperature (RT) to avoid the edge effect and then for 24 h at 37 °C, 5% CO_2_ that secure a 90% confluent cell layer.^103^

MTT assay was used to determine the maximum nontoxic concentration of the complexes on Vero cells. The cells were grown in a 96-well plate at a density of 4 × 10^4^ cells per well in 100 μL of MEM with Earle salts supplemented with 25 μg/mL gentamycin, 10% heat-inactivated FBS, 8 mM HEPES, 2 mM l-glutamine, 1× nonessential amino acids, and 1 μg/mL fungisone. The cells were incubated for 1 h at RT and then overnight at 37 °C, 5% CO_2_. When the cell layer reached around 90% confluency, the medium was complemented with serial 2-fold dilutions of complexes. Three parallels were applied for each concentration in the range of 100–0.048 μM for each complex. 10 μL of the MTT labeling reagent was added into wells at 0.5 mg/mL final concentration. The plate was incubated for 240 min at 37 °C, 5% CO_2_, and then 100 μL of the solubilization solution (10% SDS in 1 M HCl) was added to each well. Next day, the absorbance of the wells was determined by a microtiter plate reader (Labsystems Multiskan Ex 355, Thermo Fisher Scientific, Waltham, MA). The absorbance of the formazan product was measured at 540 nm.^104^

#### Investigation of the Impact of Complexes on HSV-2 Growth in Vero Cells

The Vero cells were transferred into the wells of the 96-well plate at a density of 4 × 10^4^ cells/well in 100 μL of Dulbecco’s Modified Eagle’s Medium (DMEM) (Sigma) containing 100 U/mL penicillin, 100 mg/mL streptomycin sulfate, 5% FBS, 0.25 g/mL amphotericin B, and 0.14% NaHCO_3_. Prior to infection, the cells were washed with PBS and then were incubated with HSV-2 (MOI 0.1) for 60 min at 37 °C under a 5% CO_2_ atmosphere. Then, the cells were washed with PBS again, and the culture medium complemented with serial 2-fold dilutions of the complexes to triplicate wells in the concentration range of 100–0.078 μM. The plates were incubated at 37 °C, 5% CO_2_ for 24 h. The plates were evaluated with RT-qPCR.

#### Cell Lysis and Direct Quantitative PCR

The supernatants of infected cells were removed and washed with PBS twice, and then 100 μL of high-quality ultrapure water was added to wells at the end of 24 h infection. The cells were subjected to two freeze–thaw cycles. Templates for qPCR reactions originated from mixed cell lysates. The process of PCR was described previously.^105^ Briefly, we used 5× HOT FIREPol EvaGreen qPCR Supermix (Solis Solis BioDyne, Tartu, Estonia) and HSV-2 *g*D2 gene-specific primer in a Bio-Rad CFX96 real time PCR system for the qPCR reaction. Sequences of the *g*D2 gene-specific primer pair were the following: *g*D2-F: 5′-TCAGCGAGGATAACCTGGGA-3′ and *g*D2-R 5′-GGGAGAGCGTACTTGCAGGA-3′. The annealing-extension temperature was 69 °C. The cycle where the amplification curve stepped over the baseline can correspond to the Ct value of a given sample.

### Enzyme Kinetics

Human recombinant cathepsin B was expressed in *E. coli*, recombinant cathepsin V was expressed in *Pichia pastoris*, and human recombinant cathepsin L was expressed in *E. coli*, as reported.^106–108^ For the determination of cathepsin B endopeptidase and exopeptidase activities, 100 mM phosphate buffer (pH 6.0) and 60 mM acetate buffer (pH 5.0) were used, respectively, and for the determination of cathepsin V and L activities, 100 mM acetate buffer (pH 5.5) was used. All buffers contained 0.1% PEG 8000 (Sigma-Aldrich, St. Louis, MO), 5 mM cysteine, and 1.5 mM EDTA. Prior to the assay, the enzymes were activated in the assay buffer for 5 min at 37 °C.

#### Determination of Relative Inhibition

To determine the effect of inhibitors on enzyme activity, the following substrates were used: Z-Arg-Arg-AMC (final concentration 5 μM, Z-RR-AMC; Bachem, Bubendorf, Switzerland) for cathepsin B endopeptidase activity, Abz-Gly-Ile-Val-Arg-Ala-Lys-(Dnp)-OH (final concentration 1 μM, Abz-GIVRAK-(Dnp)-OH; Bachem) for cathepsin B exopeptidase activity, and Z-Phe-Arg-AMC (final concentration 2 μM, Z-FR-AMC; Bachem) for cathepsin V and L activity. The substrate (5 μM) at a final concentration and inhibitor (5 μM) at a concentration of 50 μM were added to the wells of a black microplate. The reaction was then initiated by adding 90 μL of activated enzyme (final concentrations: 5 nM cathepsin B for its endopeptidase activity, 0.5 nM cathepsin B for its exopeptidase activity, 2.5 ng/mL for cathepsin V, and 0.02 nM for cathepsin L) in the assay buffer. The formation of fluorescent degradation products during the reaction was continuously monitored at 460 ± 10 nm with excitation at 380 ± 20 nm for Z-Arg-Arg-AMC and Z-Phe-Arg-AMC and at 420 ± 10 nm with excitation at 320 ± 20 nm for Abz-Gly-Ile-Val-Arg-Ala-Lys­(Dnp)-OH at 37 °C on a Tecan Infinite M1000 (Mannedorf, Switzerland) spectrofluorometer. All assay mixtures contained 5% (v/v) DMSO. To prevent false-positive inhibition due to the formation of compound aggregates, 0.01% Triton X-100 was used.^109^ All measurements were performed in triplicate and repeated twice. The relative inhibition was calculated using the following equation: relative inhibition (%) = 100­(1 – *v*_i_/*v*_o_), where *v*_i_ and *v*_o_ designate the reaction velocities in the presence and absence of inhibitor, respectively.

#### Determination of *K_i_* Values

Inhibition constants were calculated from the reaction velocities measured at three substrate concentrations (the substrate Z-Arg-Arg-AMC at concentrations of 60, 180, and 360 μM and the substrate Abz-Gly-Ile-Val-Arg-Ala-Lys­(Dnp)-OH at concentrations of 1, 3, and 6 μM to assess cathepsin B endopeptidase and exopeptidase activity, respectively) in the presence of seven concentrations of the inhibitor (0, 20, 40, 60, 80, 100, and 200 μM). The reaction was initiated by adding 90 μL of activated enzyme in the assay buffer at final concentrations of 5 and 0.5 nM for cathepsin B endopeptidase and exopeptidase activity, respectively, to the wells of a black microplate containing 5 μL of the substrate and 5 μL of the inhibitor. Formation of fluorescent degradation products was monitored as described above. All of the assay mixtures contained 5% (v/v) DMSO. All measurements were performed in duplicate and repeated three times. The SigmaPlot12, Enzyme Kinetics Module 1.3 was used for calculation of *K_i_* values.

### Computational Studies

Computational experiments were performed on workstations at the Department of Pharmaceutical Chemistry, Faculty of Pharmacy, University of Ljubljana, using Schrödinger Small Molecule Discovery Suite Release 2024-1 (Schrödinger, LLC, New York, USA, 2024) and Desmond/Maestro Noncommercial Distribution (Desmond v7.7, D. E. Shaw Research, New York, NY, USA, 2024).^110^ The 8B4T (resolution 1.45 Å) crystal structure of human cathepsin B (8B4T)^111^ was prepared using Protein Preparation Wizard:^112^ bond orders were assigned using CCD database, missing hydrogens were added, disulfide bonds were created, termini were capped, and missing side chains and loops were modeled with Prime.^113^ The water molecules present in the crystal structure were retained. Hydrogen bonds were automatically assigned and optimized using PROPKA (pH 7.0).^114^ Complexes **4** and **4a** were built using the Build Single Complex tool. For noncovalent docking calculations, all waters, ions, and other ligands were removed from the prepared protein, and the receptor grid was generated using Receptor Grid Generation with default settings, OPLS4 was used for parametrization of the macromolecule as well as the ligand force field and the size of active site defined by the cocrystallized ligand.^115^ Docking was performed using Glide XP software with default settings.^116^ The output poses were visualized and analyzed with Maestro software.

For MD simulations, complex **4a** was placed in the original crystal structure (with crystal waters present) in the vicinity of the catalytic triad. For the coordinatively bound **4a**-human cathepsin B complex, the aquo ligand was replaced with the ionized thiolate of Cys29, and His199 was protonated. The systems were then prepared with System Builder: TIP4P^117^ water molecules were added up to 10 Å from the protein surface to solvate the protein in an orthorhombic box, Na^+^ and Cl^–^ ions were added to neutralize the system and produce the final 0.15 M concentration, and OPLS4 ^115^ was used for parametrization of the macromolecule as well as the ligand. The default Desmond relaxation protocol (desmond_npt_relax.msj) was used for the equilibration stage followed by the production stage: 1.2 ps interval for energy, RESPA (reference system propagator algorithm) integrator with 2 fs time step, cutoff scheme at 9.0 Å, random seed, isothermal–isobaric NPT ensemble at 300 K and 1.013 bar pressure with Nose–Hoover chain thermostat, and Martyna–Tobias–Klein barostat (1 and 2 ps relaxation time, respectively, isotropic coupling). The simulation time was 100 ns with 1000 frames per trajectory being saved. The results were analyzed using built-in Desmond tools. Additional details about the analysis of the obtained trajectories are provided in Figure S14.

## Supplementary Material





## Notes

Additional experimental details and characterization data (^1^H NMR); crystallographic data; additional stability and solution chemistry data; additional enzyme inhibition; and computation study data (PDF)
Crystal structures (CIF)
