# Supplementary material for: Synthesis of a fac-Tricarbonylrhenium(I) Complex with Pyrithione, Its Physicochemical Characterization, and Assessment of Biological Effects
Source: ACS Omega. 2025 Aug 15;10(33):38272–91. doi: 10.1021/acsomega.5c06647 (PMC12391994; doi:10.1021/acsomega.5c06647)
Supplement: Supplementary file 1 [file ao5c06647_si_001.pdf]

# Supporting Information

## Synthesis of a *fac*-tricarbonylrhenium(I) complex with pyrithione, its physico-chemical characterization and assessment of biological effects

Uroš Rapuš[1], Tamás Pivarcsik[2], Ana Mitrović[3,4], Jakob Kljun[1], Anita Bogdanov[5], Gabriella Spengler[5], Anže Meden[3], Stanislav Gobec[3], Janko Kos[3,4], Éva A. Enyedy[2], Iztok Turel[1*]

[1] *Faculty of Chemistry and Chemical Technology, University of Ljubljana, Večna pot 113, SI-1000 Ljubljana, Slovenia*

[2] *Department of Molecular and Analytical Chemistry, Interdisciplinary Excellence Centre, University of Szeged, Dóm tér 7-8., H-6720 Szeged, Hungary*

[3] *Faculty of Pharmacy, University of Ljubljana, Aškerčeva cesta 7, SI-1000 Ljubljana, Slovenia*

[4] *Department of Biotechnology, Jožef Stefan Institute, Jamova 39, SI-1000 Ljubljana, Slovenia*

[5] *Department of Medical Microbiology, Albert Szent-Györgyi Health Center and Albert Szent-Györgyi Medical School, University of Szeged, Semmelweis u. 6, H-6725 Szeged, Hungary*

*Corresponding author: iztok.turel@fkkt.uni-lj.si

# Contents

Synthesis and characterization........................................2
Crystallographic data........................................8
Stability and solution chemisty........................................10
Enzyme inhibition........................................13
Computational studies........................................14

# Synthesis and characterization

## Complex 4:

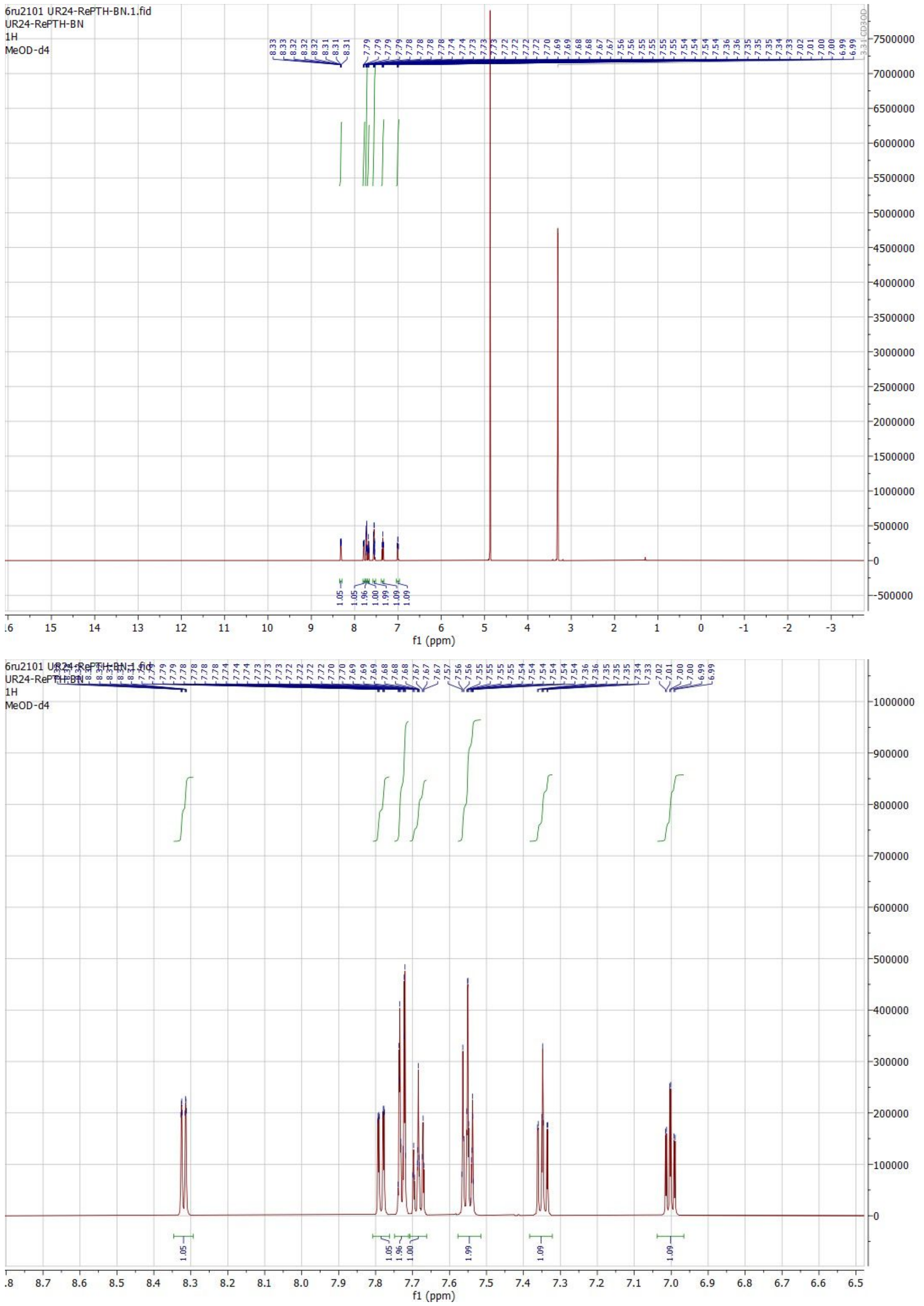


**Figure S1.** $^{1}H$ NMR spectrum of complex **4** in MeOD-$d_4$ (top) and same spectrum zoomed in aromatic region (bottom)

**Complex 1:**

In a high-pressure tube [Re(CO)$_5$Cl] (1 mol. eq.) and Napth (1 mol. eq.) were heated in 10 mL of toluene at 120 °C for one hour. The reaction mixture was cooled, and precipitate was collected with vacuum filtration. Product was washed with water and hexane and dried over night at 45 °C. **1** Yield 84.2% (118.2 mg), white solid.

$^1$H NMR (600 MHz, MeOD-*d*$_4$) δ 8.02 (dd, J = 6.6, 1.5 Hz, 1H), 7.55 (dd, J = 8.4, 2.0 Hz, 1H), 6.99 (ddd, J = 8.5, 7.1, 1.5 Hz, 1H), 6.73 (td, J = 6.9, 2.0 Hz, 1H).

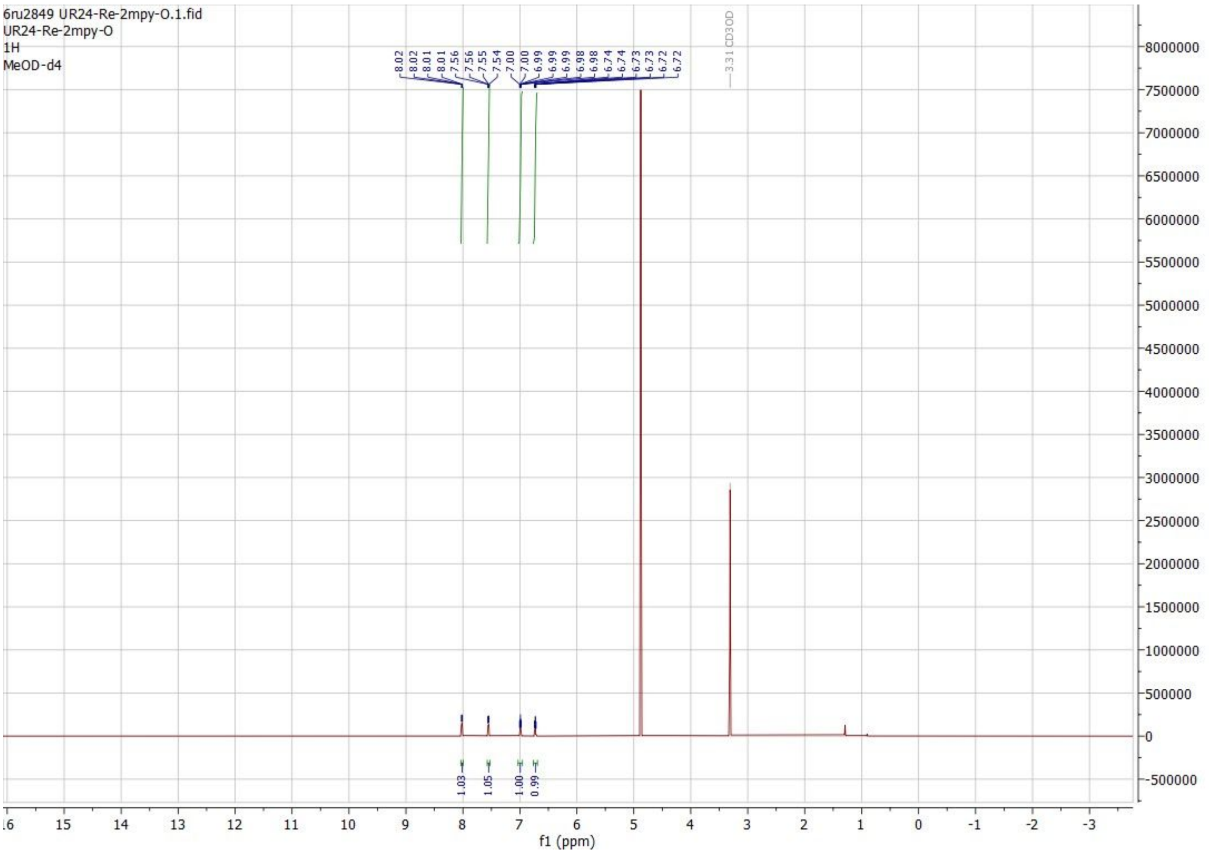


**Figure S2.** $^1$H NMR spectrum of complex **1** in MeOD-*d*$_4$.

**Complex 2:**

Precursor [Re(CO)$_3$(acetonitrile)$_2$Cl] was prepared by refluxing [Re(CO)$_5$Cl] in acetonitrile for 16 h. Reaction mixture was cooled and Napth (1 mol. eq.) was added. Reaction mixture was left stirring protected from light for another 16 h. **2** pale-yellow crystals that decompose upon removal from the solvent.

$^1$H NMR (600 MHz, CD$_3$CN-*d*$_3$) δ 8.16 (dd, J = 6.8, 0.8 Hz, 1H), 7.69 (dd, J = 8.3, 1.8 Hz, 1H), 7.28 (ddd, J = 8.4, 7.1, 1.4 Hz, 1H), 6.94 (td, J = 7.0, 1.8 Hz, 1H).

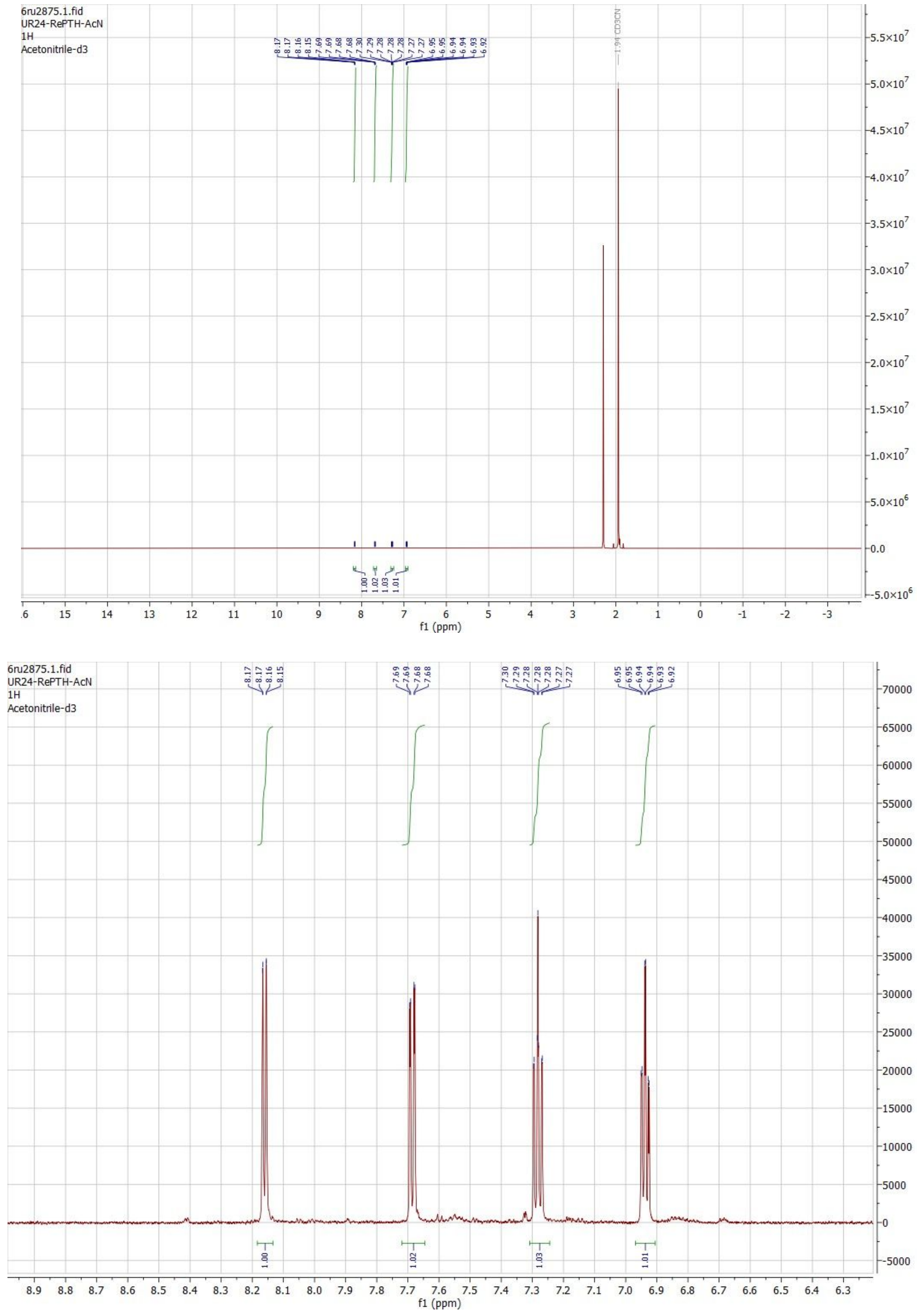


**Figure S3.** $^1$H NMR spectrum of crude complex **2** in acetonitrile-*d*$_3$ (top) and same spectrum zoomed in aromatic region (bottom).

**Complex 3:**

Synthesis of complex **3** was attempted with the same procedure as complex **4**. In the third step 2-mpyH (1.2 mol. eq.) was added to the reaction mixture and the reaction mixture was left stirring overnight protected from light. Precipitate was collected with vacuum filtration and washed with 20 mL of hexane. **3** Yield 79.8% (112.0 mg), intense-yellow solid.

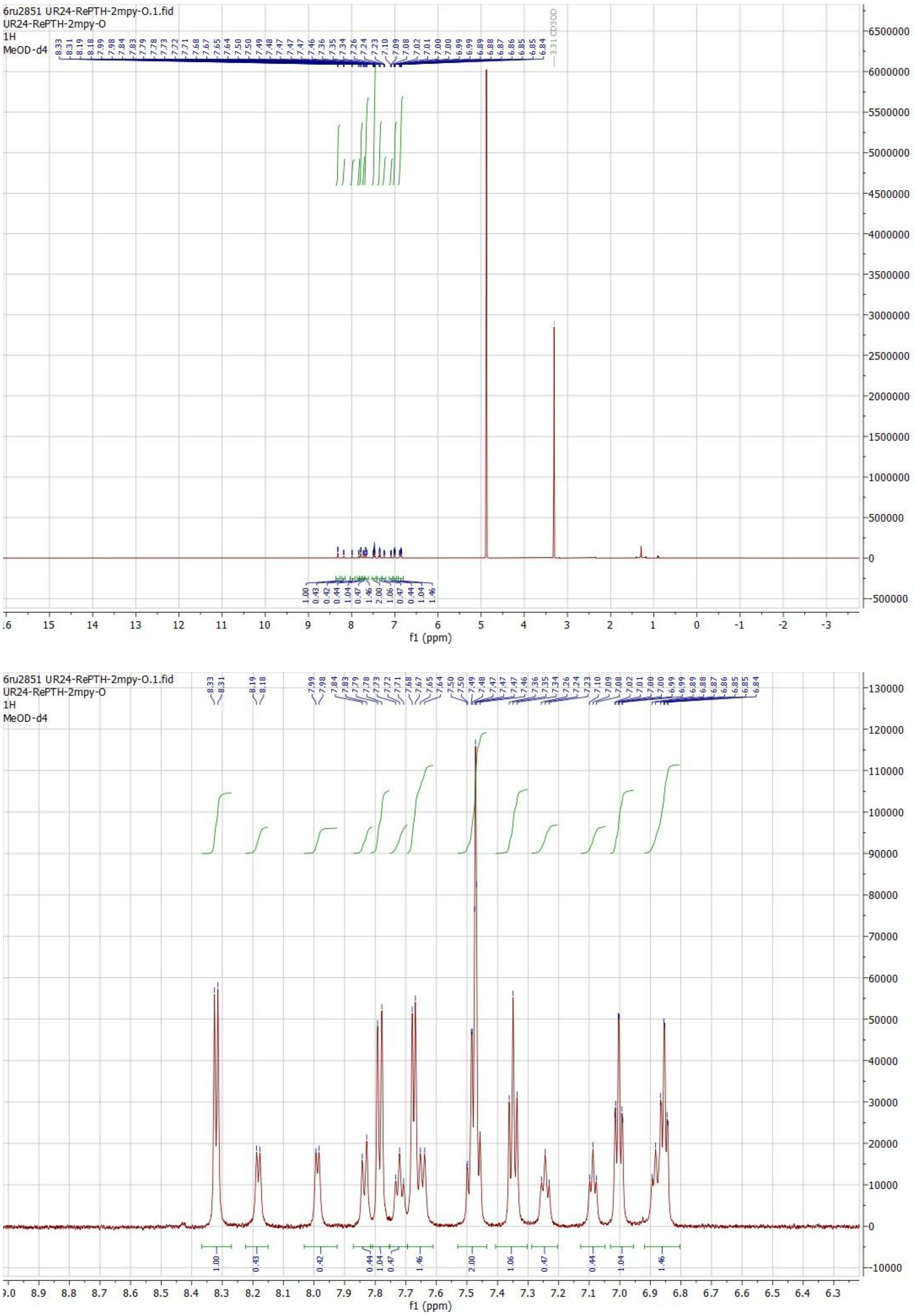


**Figure S4.** $^{1}$H NMR spectrum of crude complex **3** in MeOD-$d_4$ (top) and same spectrum zoomed in aromatic region (bottom).

**Complex 5:**

Synthesis of complex **5** was attempted with similar procedure as complex **4**. In the second step reaction was performed in $H_2O$ instead of MeOH. Overnight precipitate formed and it was collected with vacuum filtration and washed with 20 mL of hexane. **5** yellow solid, isolated product was not pure.

**Complex 6:**

Crystals of complex **6** were obtained from the NMR sample of isolated product from reaction where we tried to synthesize complex **3**.

$^{1}$H NMR (600 MHz, DMSO) δ 8.92 (s, 2H), 7.66 – 7.51 (m, 4H), 7.05 (ddd, *J* = 7.2, 5.8, 1.3 Hz, 4H), 6.78 (s, 2H).

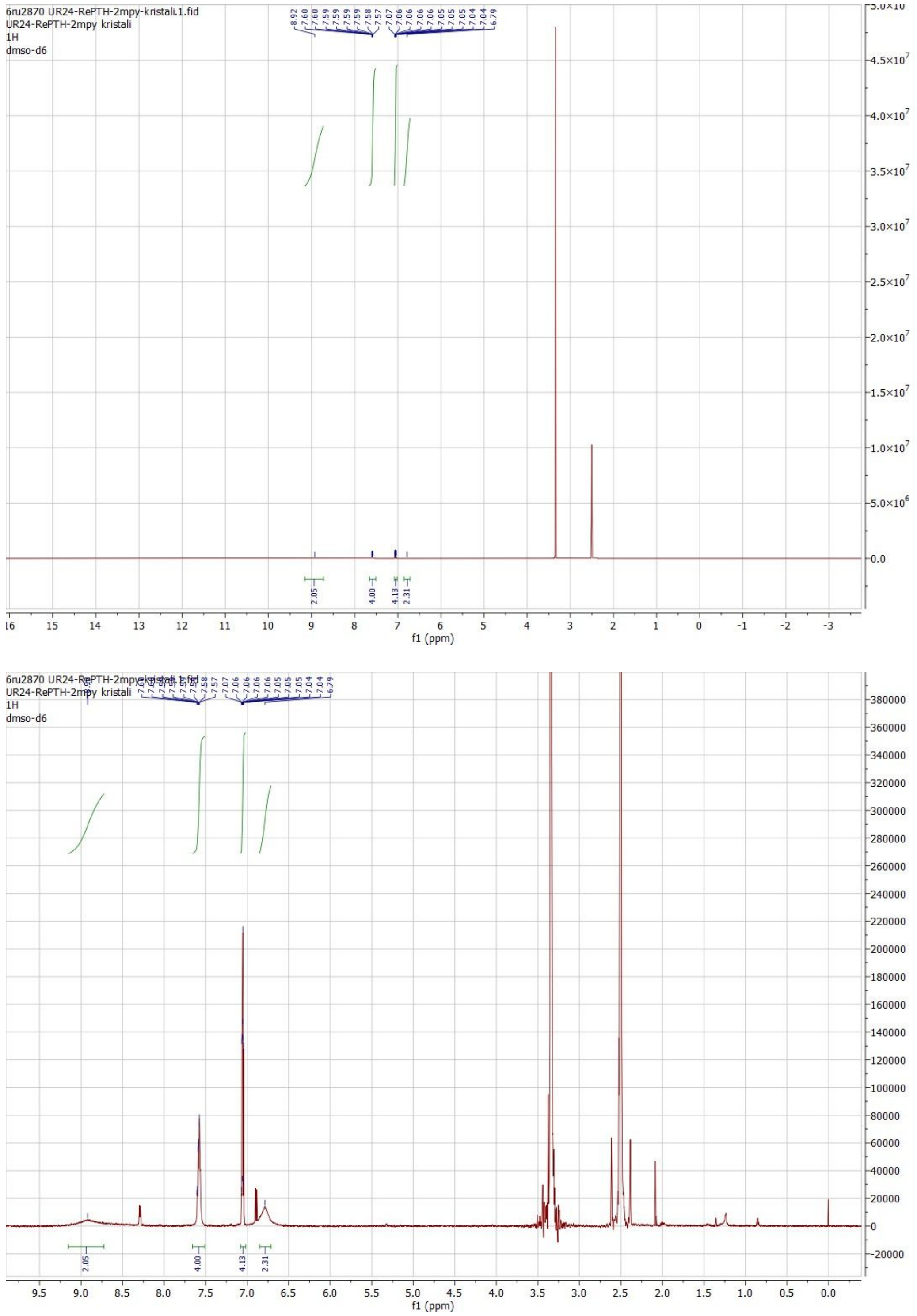


**Figure S5.** $^{1}$H NMR spectrum of crude complex **6** in dmso-$d_6$ (top) and same spectrum zoomed in (bottom).

# Crystallographic data

**Table S1.** Crystallographic data for the complex.

| Compound | **1** | **2** | **3** |
|---|---|---|---|
| CCDC No. | 2423360 | 2423361 | 2423362 |
| Sample code | moc458 | moc544 | moc545 |
| Empirical formula | $C_{16}H_8N_2O_6Re_2S_2$ | $C_{10}H_7N_2O_4ReS$ | $C_{15}H_{12}N_3O_4ReS_2$ |
| Formula weight | 760.76 | 437.44 | 548.60 |
| Temperature [K] | 150.00(10) | 150.00(10) | 150.00(10) |
| Crystal system | orthorhombic | triclinic | monoclinic |
| Space group | Pbca | P-1 | P2/c |
| a [Å] | 12.7505(5) | 5.5072(2) | 17.0424(9) |
| b [Å] | 17.0634(5) | 7.7862(3) | 7.3991(3) |
| c [Å] | 17.3107(5) | 15.1200(6) | 14.8927(7) |
| α [°] | 90 | 93.460(4) | 90 |
| β [°] | 90 | 90.303(3) | 106.712(5) |
| γ [°] | 90 | 105.549(4) | 90 |
| Volume [$Å^3$] | 3766.2(2) | 623.33(4) | 1798.63(15) |
| Z | 8 | 2 | 4 |
| $\rho_{calc}$ [g $cm^{-3}$] | 2.683 | 2.331 | 2.026 |
| μ [$mm^{-1}$] | 13.103 | 9.921 | 7.013 |
| F(000) | 2784.0 | 408.0 | 1048.0 |
| Crystal size [$mm^3$] | 0.2 × 0.03 × 0.03 | 0.3 × 0.2 × 0.1 | 0.2 × 0.1 × 0.05 |
| Radiation | Mo Kα (λ = 0.71073) | Mo Kα (λ = 0.71073) | Mo Kα (λ = 0.71073) |
| 2Θ range for data collection [°] | 4.706 to 54.964 | 5.4 to 54.96 | 5.506 to 54.96 |
| Index ranges | $-16 \leq h \leq 15$, $-21 \leq k \leq 22$, $-22 \leq l \leq 21$ | $-6 \leq h \leq 7$, $-10 \leq k \leq 10$, $-19 \leq l \leq 19$ | $-22 \leq h \leq 18$, $-6 \leq k \leq 9$, $-18 \leq l \leq 17$ |
| Reflections collected | 30328 | 13209 | 8551 |
| Independent reflections | 4290 [$R_{int}$ = 0.0713, $R_{sigma}$ = 0.0406] | 2818 [$R_{int}$ = 0.0941, $R_{sigma}$ = 0.0652] | 4010 [$R_{int}$ = 0.0299, $R_{sigma}$ = 0.0440] |
| Data/restraints/parameters | 4290/0/253 | 2818/0/122 | 4010/0/221 |
| Goodness-of-fit on $F^2$ | 1.107 | 1.252 | 1.063 |
| Final R indexes [I ≥ 2σ (I)] | $R_1$ = 0.0465, $wR_2$ = 0.1197 | $R_1$ = 0.0761, $wR_2$ = 0.2017 | $R_1$ = 0.0309, $wR_2$ = 0.0627 |
| Final R indexes [all data] | $R_1$ = 0.0580, $wR_2$ = 0.1263 | $R_1$ = 0.0792, $wR_2$ = 0.2032 | $R_1$ = 0.0413, $wR_2$ = 0.0681 |
| Largest diff. peak / hole [e $Å^{-3}$] | 4.83/-2.30 | 6.30/-4.31 | 1.59/-1.24 |

| Compound | **4** | **5** | **6** |
|---|---|---|---|
| CCDC No. | 2423363 | 2423364 | 2423365 |
| Sample code | mod29_auto | mod 169_auto | mod189_auto |
| Empirical formula | $C_{15}H_9N_2O_4ReS$ | $C_{16}H_8N_2O_8S_2Re_2$ | $C_{16}H_{12}N_3OS_3Re$ |
| Formula weight | 499.50 | 792.76 | 544.67 |
| Temperature [K] | 150.00(10) | 150.00(10) | 150.00(10) |
| Crystal system | monoclinic | triclinic | triclinic |
| Space group | P21/c | P-1 | P-1 |
| a [Å] | 15.7310(7) | 9.2949(5) | 8.3238(5) |
| b [Å] | 6.0948(2) | 10.1341(5) | 8.9855(5) |
| c [Å] | 16.3176(6) | 11.8144(5) | 12.8998(7) |
| α [°] | 90 | 95.197(4) | 80.600(5) |
| β [°] | 91.907(4) | 102.757(4) | 80.586(5) |
| γ [°] | 90 | 111.610(5) | 65.423(5) |
| Volume [$Å^3$] | 1563.62(10) | 990.80(9) | 860.70(9) |
| Z | 4 | 2 | 2 |
| $\rho_{calc}$ [g $cm^{-3}$] | 2.122 | 2.657 | 2.102 |
| μ [$mm^{-1}$] | 7.925 | 12.465 | 7.431 |
| F(000) | 944.0 | 728.0 | 520.0 |
| Crystal size [$mm^3$] | 0.1 × 0.1 × 0.05 | 0.15 × 0.1 × 0.1 | 0.3 × 0.1 × 0.1 |
| Radiation | Mo Kα (λ = 0.71073) | Mo Kα (λ = 0.71073) | Mo Kα (λ = 0.71073) |
| 2Θ range for data collection [°] | 5.182 to 54.948 | 5.132 to 54.968 | 5.014 to 54.966 |
| Index ranges | $-20 \le h \le 19$, $-7 \le k \le 7$, $-21 \le l \le 21$ | $-11 \le h \le 12$, $-13 \le k \le 13$, $-15 \le l \le 15$ | $-10 \le h \le 10$, $-11 \le k \le 11$, $-14 \le l \le 16$ |
| Reflections collected | 22999 | 11310 | 9660 |
| Independent reflections | 3569 [$R_{int}$ = 0.0860, $R_{sigma}$ = 0.0574] | 4530 [$R_{int}$ = 0.0571, $R_{sigma}$ = 0.0691] | 3944 [$R_{int}$ = 0.0583, $R_{sigma}$ = 0.0720] |
| Data/restraints/parameters | 3569/0/208 | 4530/87/271 | 3944/0/217 |
| Goodness-of-fit on $F^2$ | 1.102 | 1.037 | 1.159 |
| Final R indexes [I ≥ 2σ (I)] | $R^1$ = 0.0477, $wR_2$ = 0.1010 | $R_1$ = 0.0493, $wR_2$ = 0.1108 | $R_1$ = 0.0531, $wR_2$ = 0.1229 |
| Final R indexes [all data] | $R^1$ = 0.0832, $wR^2$ = 0.1252 | $R_1$ = 0.0730, $wR_2$ = 0.1252 | $R_1$ = 0.0593, $wR_2$ = 0.1257 |
| Largest diff. peak / hole [e $Å^{-3}$] | 3.60/-1.93 | 3.48/-1.91 | 3.69/-1.96 |

## Stability and solution chemistry

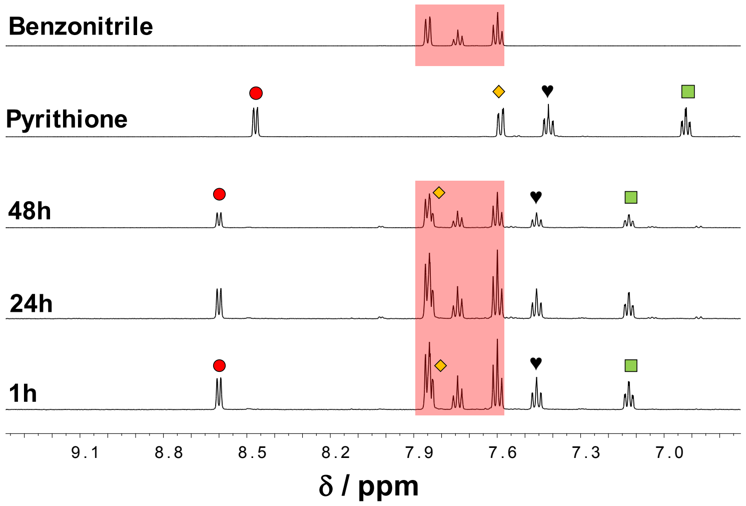


**Figure S6.** $^{1}$H NMR spectra of the complex **4** in DMSO-$d_6$ under light-exclusion conditions over time. The spectra of pyrithione and benzonitrile are also shown for comparison (peaks belonging to benzonitrile are indicated with red rectangles). Symbols for the peak assignation are shown in **Figure S7**. {$c_{complex}$ = 1 mM; $T$ = 25.0 °C} Note: in the spectrum of pyrithione a broad signal was observed at 12.2 ppm, but it could not be seen in the spectra of the complex.

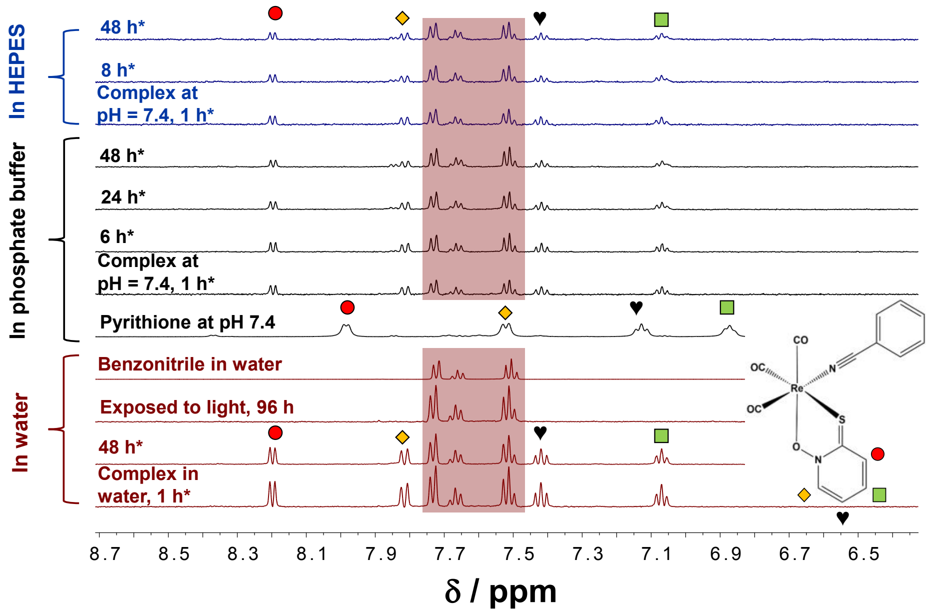


**Figure S7.** $^{1}$H NMR spectra of the complex **4** in water (pH = 6.8) (lower part), in 20 mM phosphate buffer (pH = 7.4) (middle part) and in 20 mM HEPES buffer (pH = 7.4) (upper part) over time. Symbol * denotes light-exclusion conditions. Spectra of pyrithione and benzonitrile (red rectangle) are also shown for comparison. {$c_{complex}$ = 250 μM; pH = 6.8 (water) or 7.4; 10% ($v/v$) $D_2O/H_2O$; $T$ = 25.0 °C}

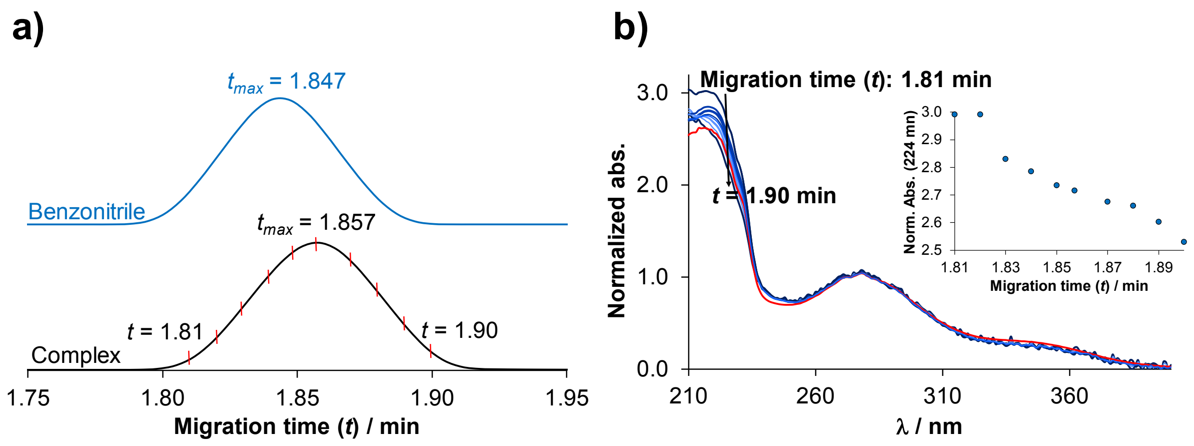


**Figure S8.** (a) Electropherograms of the complex **4** and benzonitrile under light-exclusion conditions. (b) Normalized UV-Vis spectra of the complex **4** at different migration times ($t$ = 1.81 – 1.90 min) taken from the electropherogram of the complex **4**. The time points are indicated with red lines in figure (a). UV-Vis spectrum (red line) was recorded independently using a UV-Vis spectrophotometer under the same conditions for comparison. Inserted figure shows the change in the normalized absorbance values at 224 nm as a function of migration time. Both the aqua complex and benzonitrile are neutral (at pH = 7.4), making it difficult to separate the two species, however the normalized UV-Vis spectra differ at each time point in the wavelength range 210 – 235 nm, clearly confirming that at least two species are present is solution. {$c_{complex}$ = 100 µM; pH = 7.4 (20 mM HEPES buffer); $T$ = 25.0 °C; λ = 224 nm}

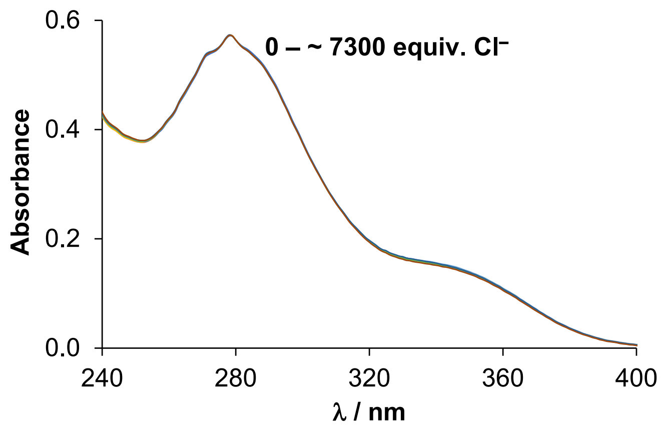


**Figure S9.** UV-Vis spectra of the complex **4** in the presence of various equivalents of chloride ions using 24 h equilibrium time. The individual samples were kept under light-exclusion conditions. {$c_{complex}$ = 60 µM; $c_{Cl^-}$ = 0 – 450 mM; pH = 7.4 (HEPES buffer); $\ell$ = 1 cm; $T$ = 25.0 °C}

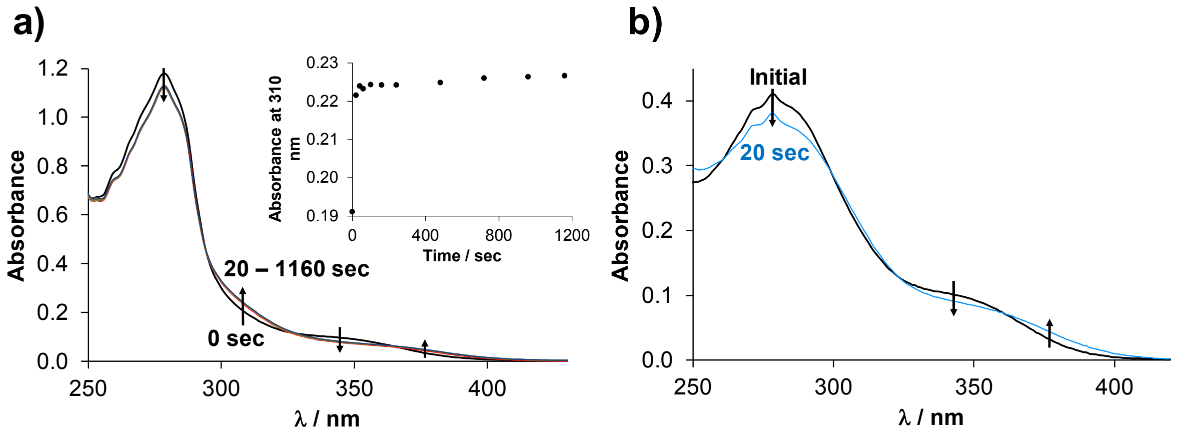


**Figure S10**. UV-Vis spectra of the complex **4** in the presence of a) HSA and b) *N*-methyl-imidazole (MIM) under light-exclusion conditions followed over time. Inserted figure shows the absorbance values at 310 nm (●) plotted against time. {$c_{complex}$ = 60 (with HSA) or 50 (with MIM) μM; $c_{HSA}$ = 30 μM; $c_{MIM}$ = 300 μM; pH ~ 6.8; ℓ = 1 cm; *T* = 25.0 °C} *Note:* The solution did not contain buffer components.

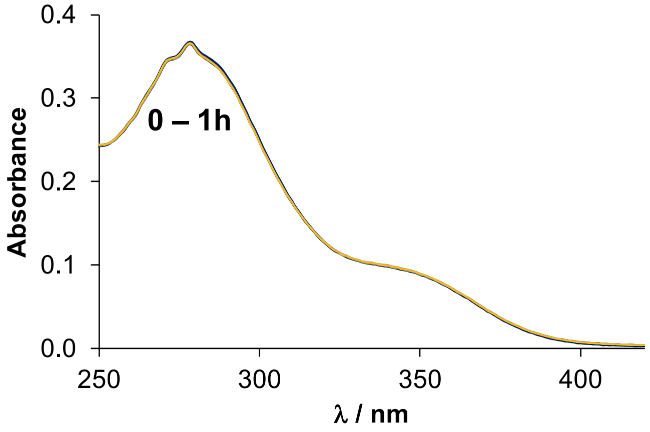


**Figure S11.** a) UV-Vis spectra of complex **4** in the presence of acetyl-cysteine (Ac-Cys) under light-exclusion conditions followed over time. {$c_{complex}$ = 50 μM; $c_{Ac\text{-}Cys}$ = 300 μM; pH = 7.4 (HEPES buffer); ℓ = 1 cm; *T* = 25.0 °C}

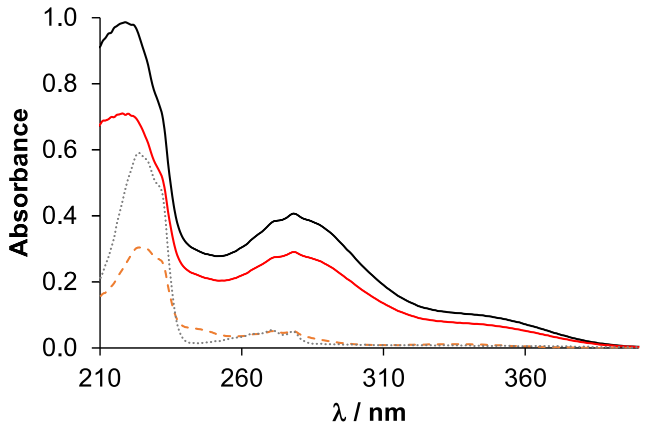


**Figure S12.** UV-Vis spectra of ultrafiltrated complex **4** in the absence (red solid line) and presence of HSA (orange dashed line) together with the nonfiltered reference spectrum (black solid line). The samples were kept under light-exclusion conditions. The spectrum of benzonitrile is also shown (grey dotted line). The complex adhered significantly to the filter (~30%), hindering the quantitative data evaluation. The spectrum recorded for the low molecular mass fraction indicates the presence of a high amount of benzonitrile. {$c_{complex/benzonitrile}$ = 50 µM; $c_{HSA}$ = 25 µM; pH = 7.4 (PBS' buffer); $\ell$ = 1 cm; $T$ = 25.0 °C}

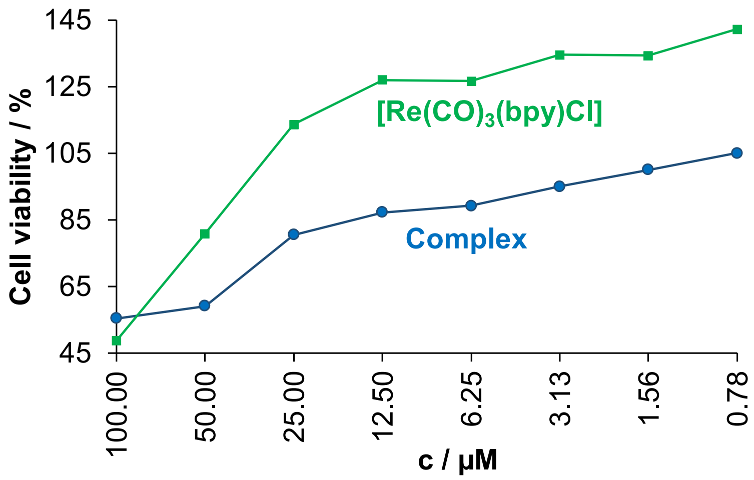


**Figure S13.** Cytotoxicity (expressed as cell viability at various complex concentration) of the complex **4** and [Re(CO)$_3$(bpy)Cl] against Vero cells. Data were taken from ref.[1] Samples were kept under light-exclusion conditions. {Incubation time: 24 h.} Reproduced from reference 1. Copyright 2024 American Chemical Society.

## Enzyme inhibition

**Table S2.** Relative inhibition of cathepsins B, L and V in % as determined at concentration 50 µM.

| | Cathepsin B | | Cathepsin L | Cathepsin V |
|---|---|---|---|---|
| | endopeptidse | exopeptidase | | |
| **Zinc pyrithione** | 8.7 ± 2.2 | 11.1 ± 2.2 | 1.3 ± 2.4 | 1.9 ± 3.0 |
| **[Ru(cym)(pth)Cl]** | 9.4 ± 1.8 | 5.4 ± 7.2 | 1.5 ± 1.5 | 1.7 ± 5.7 |

# Computational studies

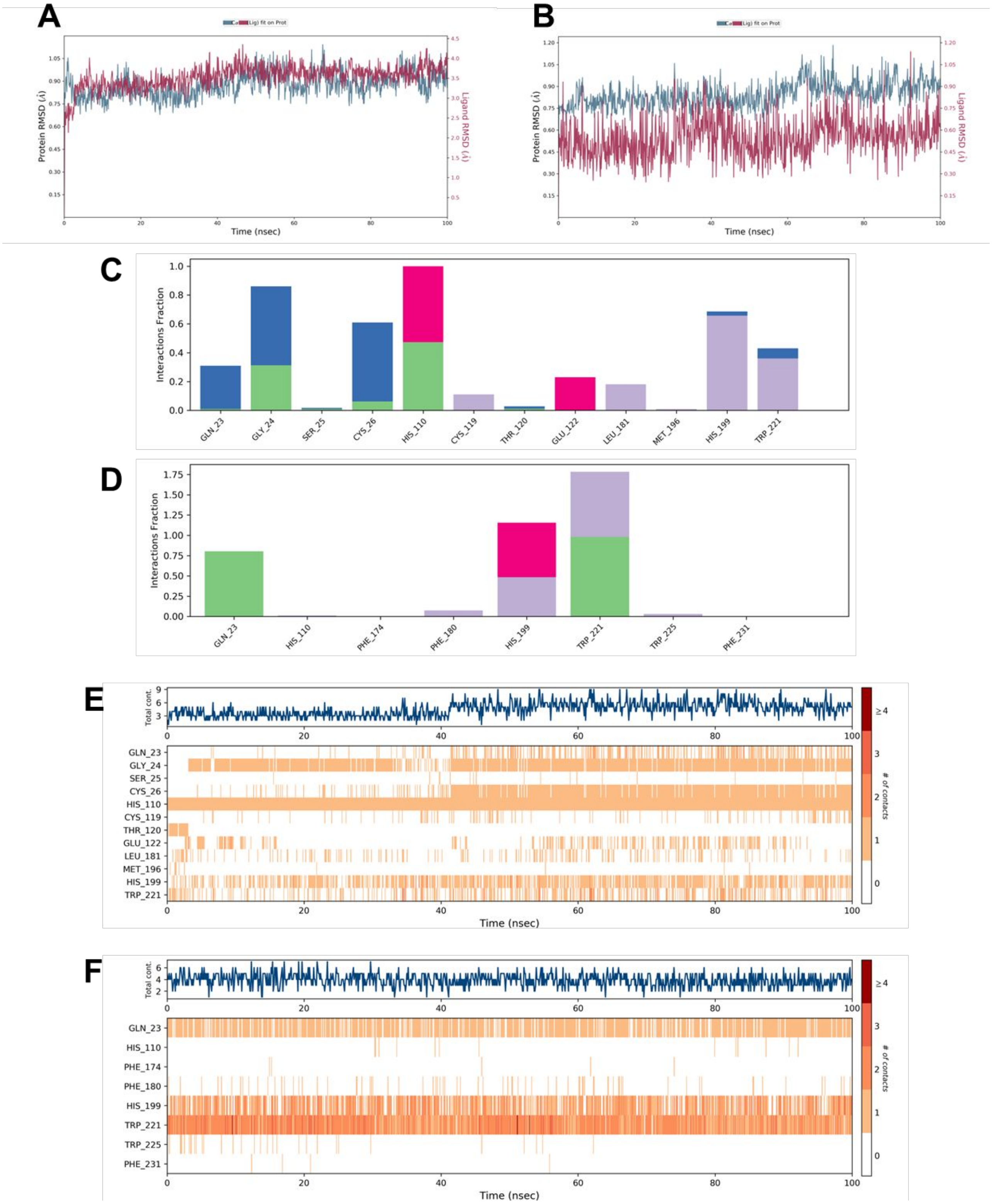


**Figure S14**. Analysis of MD trajectories for **4a**–cathepsin B reversible and covalently-bound complexes. **A**, **B** – RMSD traces over simulation time for protein (α-carbons, *vs*. frame 0, in teal) and ligand (fitted to protein backbone of frame 0, in magenta) for reversible (**A**) and covalently- (**B**) bound **4a**, respectively. **C**, **D** – ligand interactions sorted by interacting residues, for reversible (**C**) and covalently- (**D**) bound **4a**, respectively. Interactions are shown as a fraction of frames in which they occurred *vs*. the total number of simulated frames.

Hydrogen bonds are colored green, hydrophobic interactions in pink, ionic interactions in magenta, and water bridges in blue. **E**, **F** – Timeline representation of interactions presented under **C** and **D**, respectively.

# References


(1) Pivarcsik, T.; Kljun, J.; Clemente Rodriguez, S.; Cortéz Alcaraz, D.; Rapuš, U.; Nové, M.; F Várkonyi, E.; Nyári, J.; Bogdanov, A.; Spengler, G.; Turel, I.; Enyedy, É. A. Structural and Solution Speciation Studies on *fac*-Tricarbonylrhenium(I) Complexes of 2,2′-Bipyridine Analogues. *ACS Omega* **2024**, *9*, 44, 44601–44615. https://doi.org/10.1021/ACSOMEGA.4C07117.
